# Use of HSA^LR^ female mice as a model for the study of myotonic dystrophy type I

**DOI:** 10.1038/s41684-025-01506-7

**Published:** 2025-02-27

**Authors:** Marc Carrascosa-Sàez, Anna Colom-Rodrigo, Irene González-Martínez, Raquel Pérez-Gómez, Andrea García-Rey, Diego Piqueras-Losilla, Ana Ballestar, Beatriz Llamusí, Estefanía Cerro-Herreros, Ruben Artero

**Affiliations:** 1 ARTHEx Biotech, Paterna, Spain; 2 CIBER de Enfermedades Raras, Instituto de Salud Carlos III, Madrid, Spain; 3 Human Translational Genomics Group, University Institute of Biotechnology and Biomedicine, Universidad de Valencia, Burjassot, Spain; 4 Incliva Biomedical Research Institute, Valencia, Spain; 5 Present Address: Institute for Integrative Systems Biology, Consejo Superior de Investigaciones Científicas-Universitat de València, Paterna, Spain; 6 Present Address: Human Translational Genomics Group, University Institute of Biotechnology and Biomedicine, Universidad de Valencia, Burjassot, Spain; 7 Present Address: Incliva Biomedical Research Institute, Valencia, Spain; 8 Present Address: ARTHEx Biotech, Paterna, Spain

**Keywords:** Disease genetics, Disease model, Animal disease models

## Abstract

HSA^LR^ mice are the most broadly used animal model for studying myotonic dystrophy type I (DM1). However, so far, HSA^LR^ preclinical studies have often excluded female mice or failed to document the biological sex of the animals. This leaves an unwanted knowledge gap concerning the differential development of DM1 in males and females, particularly considering that the disease has a different clinical presentation in men and women. Here we compared typical functional measurements, histological features, molecular phenotypes and biochemical plasma profiles in the muscles of male and female HSA^LR^ mice in search of any significant between-sex differences that could justify this exclusion of female mice in HSA^LR^ studies and, critically, in candidate therapy assays performed with this model. We found no fundamental differences between HSA^LR^ males and females during disease development. Both sexes presented comparable functional and tissue phenotypes, with similar molecular muscle profiles. The only sex differences and significant interactions observed were in plasma biochemical parameters, which are also intrinsically variable in patients with DM1. In addition, we tested the influence of age on these measurements. We therefore suggest including female HSA^LR^ mice in regular DM1 studies, and recommend documenting the sex of animals, especially in studies focusing on metabolic alterations. This will allow researchers to detect and report any potential differences between male and female HSA^LR^ mice, especially regarding the efficacy of experimental treatments that could be relevant to patients with DM1.

## Abstract (web summary)

The authors compare muscle function, histology and molecular markers, and biochemical plasma profiles in male and female HSA^LR^ mice to identify potential sex differences in this mouse model of myotonic dystrophy type I.

## Main

Myotonic dystrophy type I (DM1) is a rare genetic disorder with autosomal dominant inheritance, which is estimated to affect 9–10 in 100,000 individuals^1^. It stems from the expansion of CTG repeats in the 3′ untranslated region (UTR) of the *DM1 protein kinase* (*DMPK*) gene^2,3^. A high number of repeats (starting at >50) produce long abnormal RNA, which becomes toxic for cell function^4^. Longer CUG repeats are associated with more severe symptoms in patients, particularly once these numbers reach the thousands^5^. Double-stranded RNA structures generated by abnormal *DMPK* transcripts with long CUG sequences result in nuclear sequestration of specific RNA-binding proteins, such as those of the muscleblind (MBNL) family, which mimic the loss of function of the sequestered proteins^6–9^. The expression of long CTG repeats in *Drosophila*^10^ and mice^11^, and MBNL1 loss of function in mice^12^, is sufficient to reproduce the DM1 condition. Typical symptoms of DM1 include progressive muscle weakness (myopathy), muscle stiffness and delayed muscle relaxation after contraction (myotonia), and progressive muscle wasting (atrophy), affecting mainly distal muscles and often appearing in adulthood^6,13^. These symptoms result in reduced strength, activity and survival, among other effects^14,15^. At a molecular level, MBNL proteins are required for proper alternative splicing of a subset of muscle, cardiac and brain transcripts^16,17^ and lead to substantial posttranscriptional modifications in many genes^6,18^. They participate in the switch from fetal and neonatal splicing forms to the adult ones necessary to develop mature muscles^19–21^. Spliceopathy is one of the main features of the DM1 muscle transcriptome. The complete miRNA content (miRNome) also exhibits changes in patients and animal models with DM1 (ref. ^18^). Indeed, miR-218 (ref. ^22^) and miR-7 (ref. ^23^) are strongly increased and decreased, respectively, in DM1 muscle biopsies and mice samples, with major consequences for muscle function. Several studies have indicated that the disease, which affects several systems, manifests differently in men and women. For example, muscle weakness in DM1 affects men more frequently, while obesity is more prevalent in women. However, these differences have not been investigated in animal models of DM1 (refs. ^15,24,25^).

So far, many different models have been developed to study DM1 (ref. ^6^). HSA^LR^ (from human skeletal actin – long repeat) mice were created in 2000 by Mankodi et al.^11^ to better understand the etiology of DM1. HSA^LR^ mice express an array of 250 CUG repeats in the 3′ UTR of the gene encoding human skeletal muscle actin. Analysis revealed that these animals develop DM1 symptoms such as myotonia and altered histology, with an increased number of central nuclei in affected myotubes, which is compatible with a pathogenic immature state^26,27^. Later studies showed that HSA^LR^ mice also replicate alternative splicing defects observed in the muscles of patients with DM1^28–30^. A review of the literature identified 41 mouse studies on HSA^LR^ (according to a PubMed search that we performed on July 2023), of which 8 documented the use of only males, and 8 the use of both male and female mice. So far, to the best of our knowledge, no study has been performed only on females. Surprisingly, most studies (61%) did not report the sex of the animals used, probably assuming that standard biomedical research is performed by default in males^31^. Investigators tend to prefer males for research, as females are thought to introduce variability through factors such as hormones^32,33^. Consequently, females are underrepresented in biomedical research^31,34^. The exclusion of HSA^LR^ females has two main consequences: (1) there are no available data regarding female-specific characteristics related to DM1 development in assays conducted using these mice, which could be critical for translation of the findings to humans; (2) excluding females increases the overall number of animals needed for each experiment and disregards valuable animals that could otherwise be used for analysis^35^.

Compared with female patients, male patients with DM1 more frequently show severe muscular disability, with marked myotonia, muscle weakness and cardiac and respiratory involvement^15,36^. The decline of upper limb performance was also investigated in a 9-year follow-up study of the natural progression of the disease, which revealed that women lost less grip strength and gross dexterity than men^37^.

Therefore, in this study, we sought to investigate whether muscle-related phenotypes were similar between male and female HSA^LR^ mice. We examined multiple key phenotypes commonly investigated in the HSA^LR^ model of DM1 to identify any notable differences between males and females that could rationalize the exclusion of females from experiments. In addition, we investigated the possibility of interaction between the sources of variation analyzed: sex × genotype. The age of the mice was also taken into consideration for statistics.

## Results and discussion

We selected a group of characteristics typically measured in DM1 studies and covering different aspects of disease alterations^11^. We analyzed muscle strength and myotonia, histological features and molecular genetics of muscle tissue, and plasma biochemical markers, with variable sample sizes (Table 1). To characterize the histological and molecular phenotypes of muscle tissue, we selected gastrocnemius and quadriceps muscle sections, because these muscles have been reported to exhibit the highest HSA transgene expression in the HSA^LR^ model^38,39^. As findings for both muscles were very similar, data for quadriceps and gastrocnemius were merged for graphs and statistical analyses (see Supplementary Dataset 1 for full details) for every individual (*n* representing the number of individuals; Table 1).

**Table 1:** Summary of the different parameters measured

Phenotype	Males WT			Females WT			Males HSA^LR^			Females HSA^LR^		
	Median	*n*	Average age	Median	*n*	Average age	Median	*n*	Average age	Median	*n*	Average age
**Age (months)**	4.57	48	–	4.50	26		4.50	52	–	4.50	29	
**HSA transgene expression (fold change)**	0.00	10	4.50	0.00	15	4.65	0.01	10	3.62	0.02	23	4.25
**Myotonia (U)**	0.00	27	4.92	0.00	3	3.85	10.00	42	4.62	10.00	11	3.60
**Weight (g)**	28.40	37	4.75	23.15	15	4.44	33.20	42	4.60	25.60	19	3.98
**Grip strength (s)**	121.62	14	4.20	122.20	15	4.44	88.73	17	4.13	85.53	19	3.98
**Strength/weight**	4.12	14	4.20	5.30	15	4.44	2.71	17	4.13	3.33	19	3.98
**Percentage of central nuclei**	4.91	21	4.48	12.47	19	4.18	28.90	37	4.63	28.73	24	4.02
***Mbnl1*** **mRNA** **(fold change)**	0.78	10	4.50	0.84	15	4.64	0.98	17	4.07	1.02	24	4.20
***Mbnl2*** **mRNA** **(fold change)**	1.15	10	4.50	1.24	15	4.64	1.02	17	4.07	0.98	24	4.20
**MBNL1 protein (fold change)**	1.00	10	4.50	1.08	15	4.64	0.98	17	4.07	0.99	24	4.20
**miR-218** **(fold change)**	0.67	10	4.50	0.45	15	4.64	1.02	17	4.07	1.01	24	4.20
***Nfix*** **exon 7 (percentage of inclusion)**	14.65	10	4.50	11.82	15	4.64	45.12	17	4.07	46.84	24	4.20
***Mbnl1*** **exon 5 (percentage of inclusion)**	3.67	10	4.50	3.73	15	4.64	32.72	17	4.07	25.88	24	4.20
***Clcn1*** **exon 7a (percentage of inclusion)**	1.91	7	4.73	2.64	14	4.65	23.21	17	4.07	20.85	24	4.20
***Atp2a1*** **exon 22 (percentage of inclusion)**	100.00	10	4.50	100.00	15	4.64	19.04	17	4.07	28.79	24	4.20
***Bin1*** **exon 11 (percentage of inclusion)**	100.00	10	4.50	100.00	15	4.64	94.35	17	4.07	95.28	24	4.20
***Cacna1s*** **exon 29 (percentage of inclusion)**	100.00	10	4.50	100.00	15	4.64	93.27	17	4.07	95.57	24	4.20
**Cholesterol (mg/dL)**	166.00	12	4.25	151.00	13	3.97	198.00	19	4.11	158.00	13	4.02
**Triglycerides (mg/dL)**	252.50	12	4.25	339.00	13	3.97	288.00	19	4.11	295.00	13	4.02
**CPK (U/L)**	1,677.00	12	4.25	345.00	13	3.97	470.00	19	4.11	257.00	13	4.02
**LDH (U/L)**	1,944.50	12	4.25	816.00	13	3.97	924.00	19	4.11	1,041.00	13	4.02
**Glucose (mg/dL)**	153.50	12	4.25	164.00	13	3.97	198.00	19	4.11	127.00	13	4.02
**AST (U/L)**	217.00	12	4.25	112.00	13	3.97	111.00	19	4.11	99.00	13	4.02

For each measure, median, sample size (*n*) and average age (months) of the animals used are given, separated by genotype (WT and HSA^LR^) and sex (males and females).

**Fig. 1:**
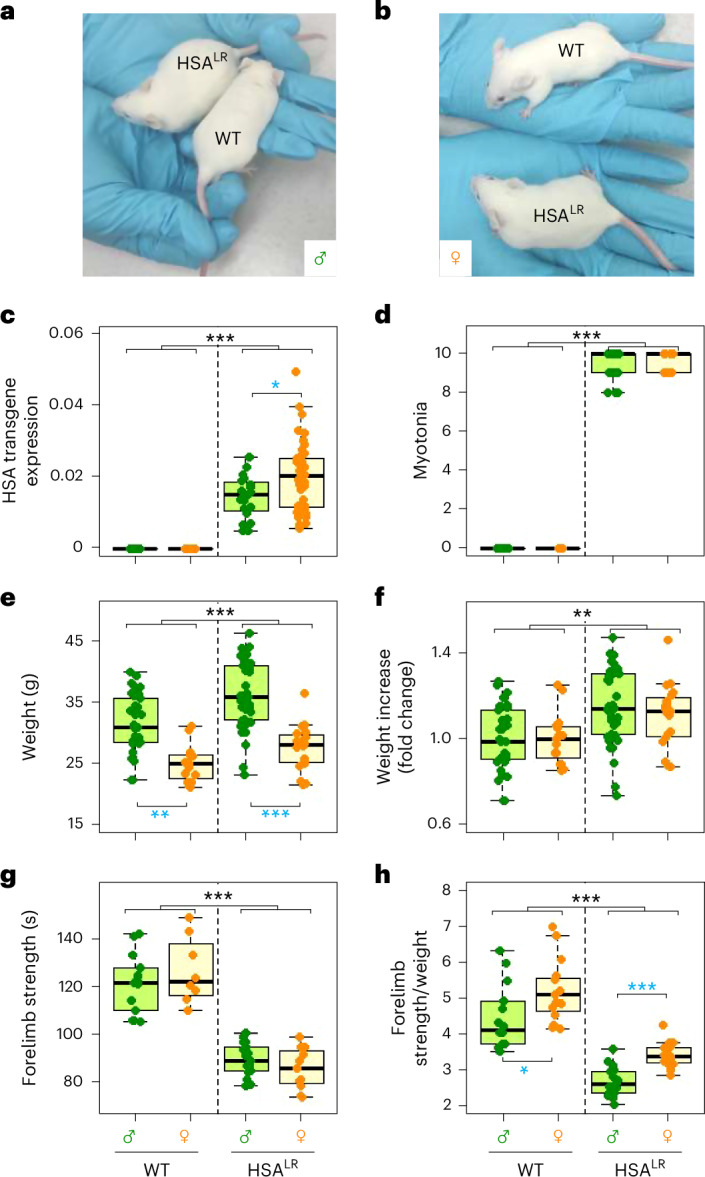
Organismal-level phenotypes. **a**,**b**, The size of male (**a**) and female (**b**) HSA^LR^ animals is increased compared with control WT. **c**, The human HSA transgene is expressed in the quadriceps and gastrocnemius of HSA^LR^ mice, but not in the muscles of WT mice. Slight but significant differences are detected in HSA transgene expression between female and male HSA^LR^ muscles (*n* = 10, *n* = 15, *n* = 10, *n* = 23, for WT males and females and HSA^LR^ males and females respectively). This observation explains the effect of sex and interaction genotype × sex observed with ANOVA (Supplementary Fig. 2). **d**, Strong myotonia is observed in HSA^LR^ animals (*n* = 27, *n* = 3, *n* = 42, *n* = 11). **e**,**f**, Weight measurements show that females are smaller than males (*n* = 37, *n* = 15, *n* = 42, *n* = 19) (**e**), and normalization to WT weight shows that weight increases consistently in both male and female HSA^LR^ animals (**f**). **g**, Forelimb strength, measured in seconds, is comparable between males and females and significantly reduced in HSA^LR^ animals (*n* = 14, *n* = 15, *n* = 17, *n* = 19). **h**, Forelimb strength normalized to weight shows significant differences between sexes and genotypes. The boxes indicate the two central quartiles, the midline represents median values and the whiskers indicate the minimum and maximum data value, excluding outliers. Statistical analysis was performed using two-way ANOVA (WT versus HSA^LR^, males versus females). Differences between WT and HSA^LR^ are represented as black asterisks. Differences between sexes were confirmed using the Wilcoxon test and are represented as blue asterisks. ***P < 0.001; **P < 0.01; *P < 0.05.

A total of 155 mice (100 male and 55 female; Fig. 1a,b) were included in this study. We first genotyped the animals to ensure that they carried the 250 CTG insertion (Supplementary Fig. 1a–d), and analyzed the HSA transgene expression (Fig. 1c), which was absent in wild type (WT) and slightly higher in female muscles (Supplementary Fig. 2a). To search for between-sex differences or possible sex × genotype interactions, we performed a two-way analysis of variance (ANOVA) for all phenotypes analyzed considering two sources of variation: genotype (WT versus HSA^LR^) and sex (males versus females). Three-way ANOVA was performed to evaluate potential effects of mouse age.

### Phenotypes of HSA^LR^ mice are similar in males and females

The expression of the HSA transgene was linked to a notable delay in skeletal muscle relaxation, a phenomenon called myotonia (Fig. 1d), which is a prominent pathological feature that also occurs in muscles of patients with DM1 (refs. ^4,40,41^). We recorded body weight and found it to be influenced by both genotype and sex (Fig. 1e and Supplementary Fig 2a). On average, HSA^LR^ animals were around 11% heavier than WT ones, and WT and HSA^LR^ males were considerably larger than females (on average, 19–23%; Table 1). The differences in size between males and females were also confirmed using the Wilcoxon test. Interestingly, when weight was normalized to the WT average weight for each sex, the fold increase due to genotype remained constant for each sex (Fig. 1f). Despite the size differences between males and females, we noted no significant differences between sexes in forelimb strength determined by the grip resistance test (Fig. 1g and Methods); however, there was an apparent reduction of around 30% in grip strength in HSA^LR^ mice compared with WT mice (Supplementary Fig. 2a). Many strength measurements used to study muscle dystrophy in mice^42^ are normalized to body weight. In Fig. 1h, we show the ratio between grip strength and weight. As body weight is a sex-dependent characteristic, when we normalized the grip measures to the weight, the resulting data were also sex dependent. Importantly, we detected no interaction between sex and genotype in this set of in vivo measurements (Supplementary Fig. 2a). Further analysis considering the age of the animals showed that the weight, strength and myotonia of animals significantly increased in older animals (Supplementary Fig. 2b–h).

On average, we observed that males were larger than females, and that weight gain associated with the HSA^LR^ genotype affected both sexes proportionally. Previous studies have examined grip strength in male and female HSA^LR^ mice, although they did not provide detailed information on the calculation methods used or whether they observed any differences between sexes^43–46^. In our study, we found no significant differences in grip strength between males and females. Grip strength was reduced in HSA^LR^ animals, but there was no notable difference between sexes. We further confirmed this observation using a new different set of animals of intermediate ages, measuring both hindleg and foreleg strength (Supplementary Fig. 3). Many different devices are available for evaluating the strength of laboratory animals, some of which can be affected by the sex or weight of the animals. When measuring strength alterations, the devices require prior testing to determine whether males and females perform differently in different settings. In vivo phenotypes can therefore be studied in both male and female HSA^LR^, always taking animal weight and weight-derived parameters into account.

### Central nuclei and histology

**Fig. 2:**
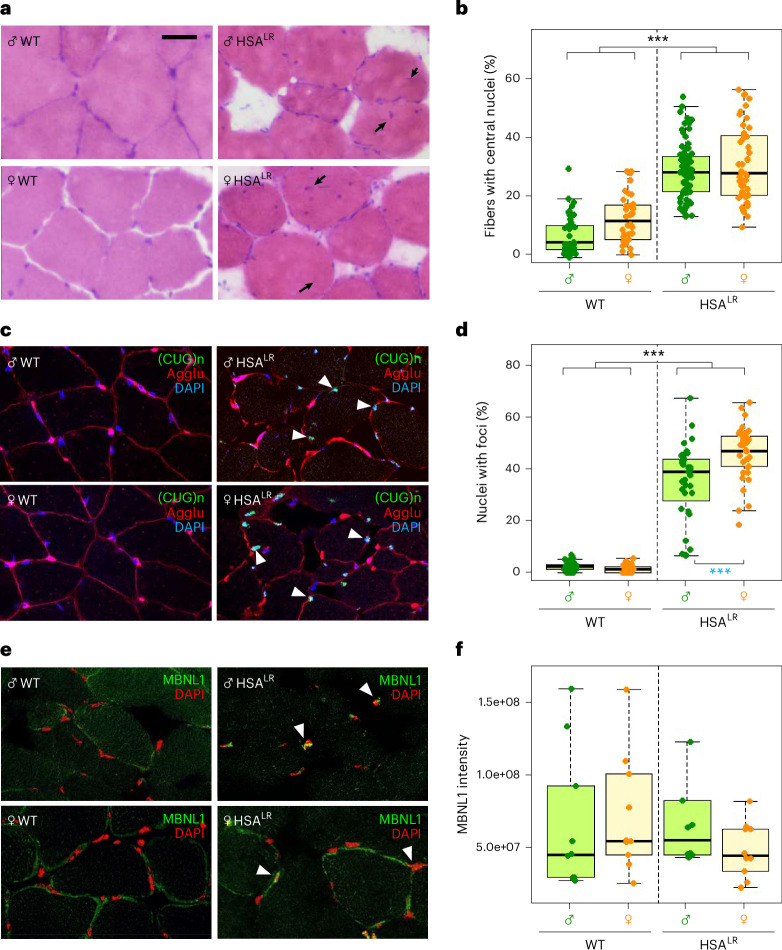
Muscle histology in male and female WT and HSA^LR^ mice. **a**, Representative bright-field microscopy pictures (200× magnification; scale bar, 50 µm) of hematoxylin–eosin staining of quadriceps sections showing an increase in the amount of central nuclei (black arrows) in HSA^LR^ mice. **b**, The percentage of fibers with central nuclei is dramatically increased in HSA^LR^ muscles (quadriceps and gastrocnemius combined; WT males *n* = 21, WT females *n* = 19, HSA^LR^ males *n* = 37, HSA^LR^ females *n* = 24). **c**, Representative confocal images (gastrocnemius; 400× magnification) showing the detection of CUG repeats (green) and agglutinin (red) as a marker of cell membrane. **d**, The percentage of nuclei with foci in the muscles studied (quadriceps and gastrocnemius combined). The individual quantification of each slide is shown (seven slides per muscle per mouse; WT males *n* = 5, WT females *n* = 5, HSA^LR^ males *n* = 5, HSA^LR^ females *n* = 5). **e**, Representative confocal images (gastrocnemiu*s;* 400× magnification) where MBNL1 was stained (green). **f**, MBNL1 fluorescence was quantified (quadriceps and gastrocnemius combined). The mean quantification of the slides is shown (seven slides per muscle per mouse; WT males *n* = 5, WT females *n* = 5, HSA^LR^ males *n* = 5, HSA^LR^ females *n* = 5). The boxes indicate the two central quartiles, the midline represents median values and the whiskers indicate the minimum and maximum data value, excluding outliers. Statistical analysis was performed using two-way ANOVA (WT versus HSA^LR^, males versus females). Differences between WT and HSA^LR^ are represented as black asterisks. Differences between sexes were confirmed using the Wilcoxon test and are represented as blue asterisks. ***P < 0.001; **P < 0.01; *P < 0.05.

We prepared quadriceps and gastrocnemius muscle sections (Supplementary Fig. 4a) from male and female mice of both genotypes (Table 1) and used hematoxylin and eosin staining to reveal muscle fibers and their nuclei (Fig. 2a and Supplementary Fig. 4b). Nuclei are known to migrate to the periphery of the myotubes during differentiation, and the presence of centralized myonuclei in adults is a sign of myofiber immaturity^26^. We obtained the percentage of muscle fibers with centrally located nuclei from at least 100 fibers per mouse in both quadriceps and gastrocnemius (before combining data from both muscles), which was dramatically increased in HSA^LR^ animals compared with WT (Fig. 2b and Supplementary Fig. 4c). The myofiber immaturity phenotype is clearly visible in our sample (Fig. 2a,b and Supplementary Fig. 4b), as previously described^44,45,47^ and in agreement with the original description of HSA^LR^ mice in the report of Mankodi et al.^11^. As expected, age had a significant effect on the percentage of fibers with central nuclei (Supplementary Fig. 4d,e). The shape of muscle fibers seemed altered in HSA^LR^ mice, with typical increased fat infiltration (Fig. 2a and Supplementary Fig. 4b), as observed elsewhere^48–50^. However, we could not confirm differences in the cross-sectional area of gastrocnemius fibers among groups, neither between sexes nor between genotypes (Supplementary Fig. 4f). The accumulation of transcripts with long CTG repeats and muscleblind 1 (MBNL1) proteins into the nucleus form so-called foci, which we detected in the muscles of HSA^LR^ mice using a (CUG)_*n*_ probe (Fig. 2c). We also observed aggregates of the abnormal mRNA in the nucleus in HSA^LR^ muscles (but rarely in WT), which were slightly more abundant in females (Fig. 2d and Supplementary Fig. 4c), possibly corresponding to the slightly higher HSA transgene expression observed by quantitative reverse-transcription polymerase chain reaction (qRT–PCR) in HSA^LR^ females (Fig. 1c). As expected, MBNL1 protein seemed to accumulate in the nuclei in muscles of HSA^LR^ mice (Fig. 2e), as a result of being sequestered by the CUG repeats, while in WT muscles, MBNL1 seemed to be distributed mostly in the cytoplasm. Quantification of Mblnl1 fluorescence in the subset of samples analyzed revealed high variation from one sample to another (Fig. 2f), not allowing any conclusion on potential differences. We conclude that, despite the high level of variation found among muscles using this technique, we clearly observed a different localization of the MBNL1 protein in HSA^LR^ muscles compared with WT, presumably forming the foci.

### *Mbnl1* and *Mbnl2* expression is similarly altered in HSA^LR^ males and females

**Fig. 3:**
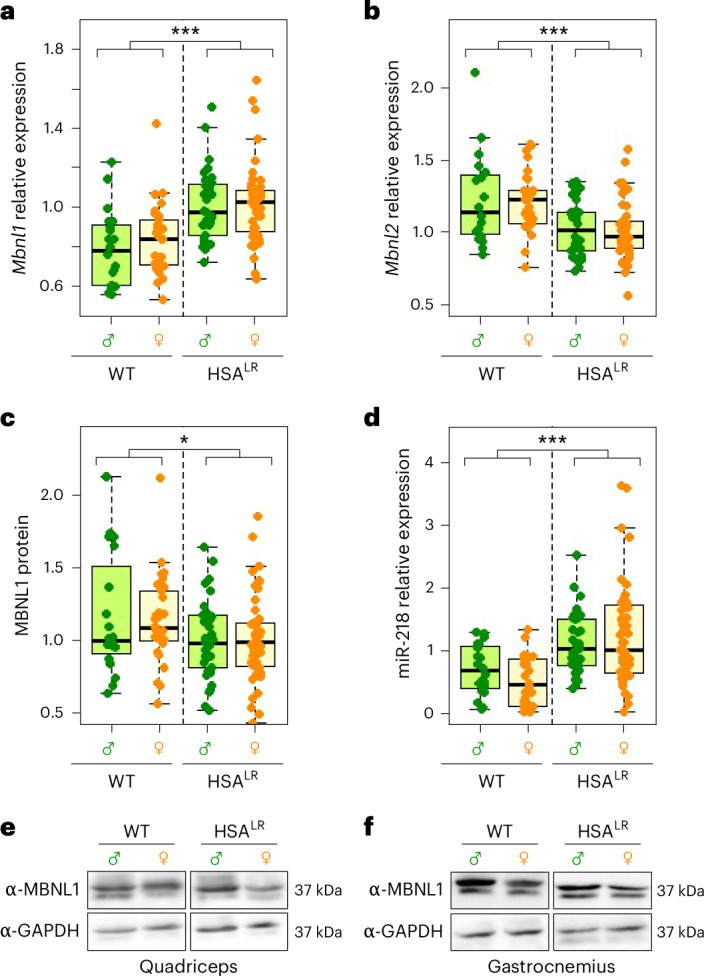
Muscleblind family gene expression analysis. **a**, *Mbnl1* gene expression, measured by qRT–PCR, is significantly increased in HSA^LR^ muscles compared with WT. **b**, By contrast, *Mbnl2* expression is reduced in HSA^LR^ muscles. **c**, MBNL1 protein levels, as measured by quantitative dot blot, show a significant decrease in HSA^LR^ muscles compared with WT. **d**, miR-218 levels are increased in the muscles of HSA^LR^ animals compared with WT. The boxes indicate the two central quartiles, the midline represents median values and the whiskers indicate the minimum and maximum data value, excluding outliers. **e**,**f**, Representative western blot membranes depicting MBNL1 protein levels in quadriceps (**e**) and gastrocnemius (**f**). Statistical analysis was performed using two-way ANOVA (black asterisks) ***P < 0.001; *P < 0.05. Sample sizes for the whole figure: *n* = 10, *n* = 15, *n* = 17 and *n* = 24 for WT males and females and HSA^LR^ males and females, respectively. Quadriceps and gastrocnemius were analyzed for each individual.

In patients with DM1, the high number of CUG repeats in the *DMPK* transcript produces aggregates with MBNL1 in the nucleus, called foci, which can be detected microscopically^9,51^. MBNL1 sequestration in these ribonuclear structures mimics protein loss of function, which is reported to have severe consequences for muscle development^12,52^. We measured the expression of *Mbnl1* (Fig. 3a) and *Mbnl2* (Fig. 3b)—two genes involved in DM1 phenotype^53,54^—by qRT–PCR in WT and HSA^LR^ muscles (quadriceps and gastrocnemius). *Mbnl1* expression was upregulated in HSA^LR^ muscles, as previously shown^22^, probably as part of a response of the tissue to restore normal *Mbnl1* levels. By contrast, we found significantly lower levels of *Mbnl2* in HSA^LR^ tissue compared with WT, as previously reported^55^; however, the cause of this reduction is unclear. Previous studies have also reported increased or unchanged *Mbnl2* expression in the muscles of these mice^22,45^. Despite the increase in *Mbnl1* transcription, we observed no increase in MBNL1 protein (Fig. 3c,e,f), suggesting that its translation or protein stability could be somehow compromised. In fact, we observed slightly, but significantly, reduced MBNL1 levels in HSA^LR^ animals, which has been observed before in HSA^LR^ and DM1 cells^50,56^.

The miRNome is also altered in muscles of HSA^LR^ mice and patients with DM1 (refs. ^18,23^). It has been reported that miR-218 is upregulated and could impinge on *Mbln1* and *Mbln2* expression^22,56^. We found that miR-218 was consistently upregulated in the muscles of HSA^LR^ male and female mice (Fig. 3d). miR-218 inhibition of *Mbnl1* mRNA translation^56^ could explain why the higher *Mbnl1* transcript levels in HSA^LR^ mice were not reflected in MBNL1 protein levels (Fig. 3a,c).

Statistical analysis indicated that *Mbnl1* and *Mbnl2* expression, MBNL1 expression and miR-218 expression were similar between males and females (Supplementary Fig. 5a), as there was no influence of sex or interaction between genotype and sex for any of these parameters.

There is a wide between-study variation in MBNL1 and MBNL2 levels. Although differences in the age of the animals included in the studies could explain this variation, we did not detect any influence of age on *Mbnl1*, MBNL1 and miR-218 levels (Supplementary Fig. 5b,d,e). In the case of *Mbnl2*, we detected increased expression due to age only in WT muscles, while *Mbnl2* expression remained constant in HSA^LR^ mice (Supplementary Fig. 5c,f). Determining how these genes are regulated and how this variability is controlled could be an interesting direction for future research. Conceivably, increasing sample sizes and widening the age range analyzed could contribute to a better understanding of this variability.

### Defects in alternative splicing are precisely replicated in HSA^LR^ females

**Fig. 4:**
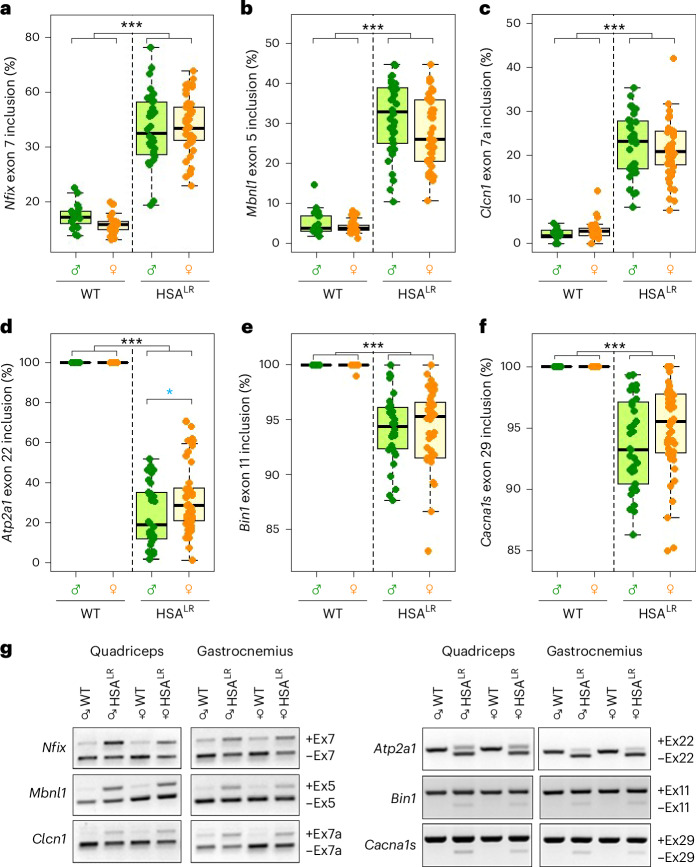
Alternative splicing defects. Comparison of typical missplicing events in HSA^LR^ muscles in males (green) and females (orange). **a**–**f**, Percentages of abnormal inclusion of exon 7 in *Nfix* (**a**), exon 5 in *Mbnl1* (**b**) and exon 7a in *Clcn1* (**c**) transcripts and abnormal exclusion of exon 22 in *Atp2A1* (**d**), exon 11 in *Bin1* (**e**) and exon 29 in *Cacna1s* (**f**). The boxes indicate the two central quartiles, the midline represents median values and the whiskers indicate the minimum and maximum data value, excluding outliers. **g**, Representative images of agarose gel electrophoresis from semiquantitative PCR determinations of the indicated alternative exons (Ex). Statistical analysis was performed using two-way ANOVA (black asterisks). Minor effects are observed due to the sex of the animals, which are confirmed only in *Atp2a1* using the Wilcoxon test (blue asterisks) ***P < 0.001; **P < 0.01; *P < 0.05. Sample sizes for the whole figure: *n* = 10, *n* = 15, *n* = 17 and *n* = 24 for WT males and females and HSA^LR^ males and females, respectively, except *Clcn1* (*n* = 7, *n* = 14, *n* = 17 and *n* = 24 for WT males and females and HSA^LR^ males and females, respectively). Quadriceps and gastrocnemius were both analyzed for each individual.

Together with other RNA-binding proteins, Muscleblind proteins are involved in the alternative splicing and polyadenylation processing of numerous genes in muscle cells from patients with DM1 (refs. ^17,21,57–61^) and HSA^LR^ muscle tissue^55,56,62^. When analyzing the quadriceps and gastrocnemius of the mice, we investigated whether the splicing pattern of a few chosen genes with clearly documented alternative splicing defects in DM1 and HSA^LR^ males were also altered in female HSA^LR^ mouse muscles^57,63,64^ (Fig. 4). Three of these genes are characterized by increased exon inclusion (*Nfix*, *Mbnl1* and *Clcn1*) and three others by increased exon exclusion (*Atp2a1*, *Bin1* and *Cacna1s*).

*Nfix*, which encodes a transcription factor involved in extracellular matrix remodeling during myogenesis, presents an abnormal inclusion of exon 7 in 68% of DM1 muscle samples^45,61,65^, in accordance with previous observations in HSA^LR^ mice^45,66^. We found inclusion of exon 7 of *Nfix* in HSA^LR^ mice to be around 50%, compared with 13–15% in control samples (Fig. 4a,g), with no differences between males and females (Supplementary Fig. 6a). *Nfix* exon 7 inclusion is controlled by MBNL2 (ref. ^60^), suggesting a possible link between the observed reduction in *Mbln2* expression (Fig. 3b and Supplementary Fig. 5a) and *Nfix* missplicing. Exon 5 inclusion in *Mbnl1* transcripts, an event that rarely occurs in control samples, was dramatically increased (around 30%) in HSA^LR^ animals compared with control ones (Fig. 4b,g). With ANOVA, we detected a very weak influence of sex in this phenotype (Supplementary Fig. 6a), which was not confirmed with the Wilcoxon test. *Clcn1* encodes a chloride channel regulating the electric excitability of the skeletal muscle membrane. The anomaly in the splicing pattern of *Clcn1* mRNA underlies the prominent myotonia found in muscles of patients with DM1 and HSA^LR^ mice^45,67–69^. We observed an increase of exon 7a inclusion in HSA^LR^ samples, on average 20–25% more than controls, in both males and females (Fig. 4c,g).

Abnormal exclusion of *Atp2a1* exon 22, *Bin1* exon 11 and *Cacna1s* exon 29 are typical signatures in DM1 and HSA^LR^ muscle transcriptome. *Atp2a1*, also known as *SERCA1*, encodes a sarcoplasmic reticulum calcium-channel protein essential for proper muscle contraction^64–66^. In our study, exon 22 was always included in control animals, while in HSA^LR^ muscle samples only 20–30% of the transcripts retained this exon (Fig. 4d,g). Compared with the impact of the genotype, ANOVA (Supplementary Fig. 6a) revealed modest differences between males and females HSA^LR^ that we confirmed using the Wilcoxon test. *Bin1* encodes a protein necessary for the biogenesis of muscle T tubules, which are specialized skeletal muscle structures essential for excitation–contraction coupling; exon 11 exclusion could contribute to the presence of central nuclei in HSA^LR^ mice. We observed a small but significant decrease of exon 11 inclusion in HSA^LR^ mice (Fig. 4e,g and Supplementary Fig. 6a), similar to what has been previously described in muscular biopsies from patients with DM1 (ref. ^70^). Finally, *Cacna1s* exon 29 exclusion enhances L-type Ca^2+^ channel conductance and voltage sensitivity in mouse muscle fibers. While exon 29 was always included in control mice, we observed an increase in *Cacna1s* missplicing in HSA^LR^ animals (Fig. 4f,g) consistent with published data^71^.

Interestingly, data analysis showed that the age of the animals was an important source of variation for splicing pattern (Supplementary Fig. 6b–h), with older HSA^LR^ animals displaying splicing levels closer to a WT situation, particularly older females, with *Nfix* being the only exception. In this study, males kept similar values of abnormal inclusion or exclusion of analyzed exons for all ages, but this finding could change for animals older than 6 months. Our observations fit with previously published data^59,65^ that show that the HSA^LR^ missplicing degree in the muscles of young animals is stronger than in old ones for *Clcn1* and *Atp2a1*, among other genes. Some investigators have suggested that splice switching from fetal to postnatal patterns is temporarily delayed in HSA^LR^ mice, but not prevented^72^. In our hands, the missplicing events were comparable for males and females mostly in young animals (around 3 months) and differences appeared later with age. These differences could be considered negligible owing to the remarkable differences between genotypes, which are still excellent molecular markers of the disease.

### Biochemical markers in blood plasma

**Fig. 5:**
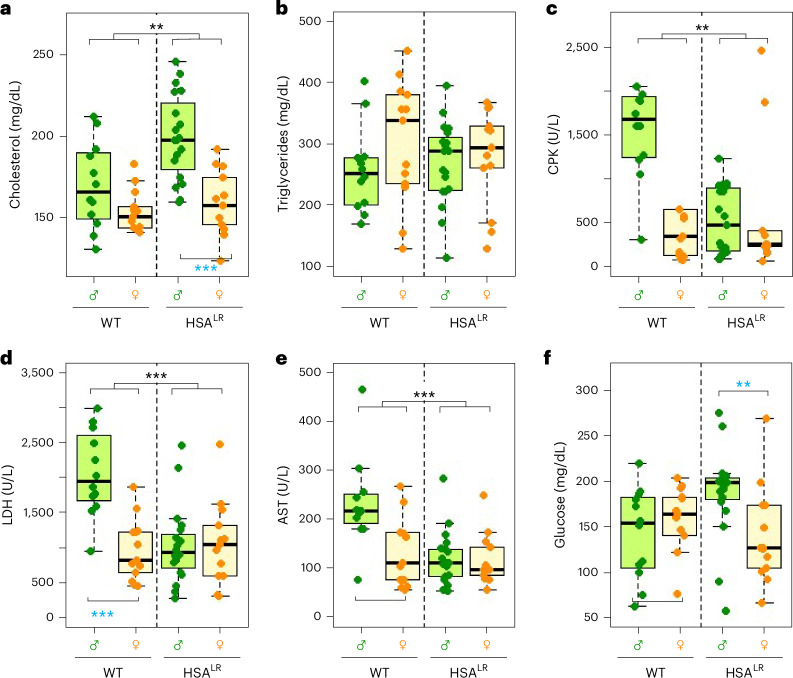
Plasma biochemical parameters. **a**–**f**, Plasma levels of cholesterol (**a**), triglycerides (**b**), CPK (**c**), LDH (**d**), AST (**e**) and glucose (**f**) were analyzed in WT and HSA^LR^ mice. The boxes indicate the two central quartiles, the midline represents median values and the whiskers indicate the minimum and maximum data value, excluding outliers. Statistical analysis was performed using two-way ANOVA (black asterisks). Detected differences between sexes were confirmed using the Wilcoxon test (blue asterisks) ***P < 0.001; **P < 0.01; *P < 0.05. Sample sizes for the whole figure: *n* = 12, *n* = 13, *n* = 19 and *n* = 13 for WT males and females and HSA^LR^ males and females, respectively.

Biochemical analysis revealed significantly increased plasma cholesterol in HSA^LR^ animals compared with WT (Fig. 5a and Supplementary Fig. 7a). Interestingly, cholesterol was significantly lower in females than in males, particularly in HSA^LR^ animals. Triglyceride levels were highly variable and did not present significant alterations among groups analyzed (Fig. 5b and Supplementary Fig. 7a). Creatine phosphokinase (CPK), lactate dehydrogenase (LDH) and aspartate transferase (AST) levels showed very similar patterns (Fig. 5c–e), with very high values in WT males. Statistically significant differences were observed between males and females in WT animals for LDH, with a similar pattern being observed for CPK, and AST levels. Notably, for all these parameters, differences between sexes were absent in HSA^LR^ mice (Fig. 5c–e and Supplementary Fig. 7a). A different pattern was observed for glucose levels (Fig. 5f and Supplementary Fig. 7a), showing especially low values in control males.

Overall, we found statistically significant differences between WT and HSA^LR^ for cholesterol, CPK, LDH and AST values. Abnormally low LDH circulating levels are rare but considered not harmful^73^. Taken together, we observed a slight increase in cholesterol levels, and a reduction in CPK, LDH and AST levels, which affected mostly HSA^LR^ males. Further work is needed to gain a greater insight into the role of these enzymes in DM1. Plasma biochemical features are intrinsically variable in patients with DM1. Nevertheless, it is well documented that these patients show increased body weight, characterized by adipose tissue expansion, despite muscle fiber atrophy^4,74,75^. A sedentary lifestyle and related muscle weakness contribute to the onset of metabolic syndrome in these patients. Patients with DM1 often exhibit increased plasma cholesterol and triglyceride levels, insulin resistance and an increased risk of developing metabolic syndrome^76–81^. Patients with DM1 often develop hyperglycemia^76,79^. Sex differences have been reported for several metabolic features in patients with DM1 (refs. ^79,81^). So far, the biochemical profile of HSA^LR^ mice has not been described in detail, but previous studies have shown that they develop insulin resistance to some degree and often present altered plasma lipid profile^42^. We showed above that HSA^LR^ animals are generally heavier than control (Fig. 1a,b,e,f), with an increase in adipocytes and infiltrated fat in muscle composition (Fig. 2a and Supplementary Fig. 4b), similar to the muscles of patients with DM1 (refs. ^76,82^). HSA^LR^ mice also exhibit features compatible with insulin resistance, such as increased cholesterol in both sexes and higher glucose levels in circulating plasma of males. LDH variations have not been previously characterized in HSA^LR^ mice, but it would be interesting to further determine to what extent they are linked to impaired glucose metabolism in muscle. CPK catalyzes the conversion of creatine to create phosphocreatine and has been reported to be slightly elevated in the blood of patients with DM1 (ref. ^41^). Unlike the findings from patients with DM1, we found that CPK average levels in HSA^LR^ mice were consistently reduced in a genotype-dependent manner (Fig. 5c and Supplementary Fig. 7a). Both CPK and LDH leak from damaged muscle and can be used as an indicator of muscular metabolic state^83^. Abnormal liver tests in patients with DM1 (ref. ^84^) show high AST levels, while we observed reduced AST levels for HSA^LR^ mice compared with WT mice (Fig. 5e). The implications of CPK correlation with AST and LDH levels in HSA^LR^ mice and the role of muscle metabolism in plasma glucose levels are unclear and warrant further investigation.

When taking the age of the animals into consideration for the analysis (Supplementary Fig. 7b–h), we also observed that triglycerides and LDH tend to increase and glucose tends to decrease with age (Supplementary Fig. 7c,e,g,h), with the trend being stronger in males. Cholesterol, CPK and AST did not show a clear correlation with age (Supplementary Fig. 7b,d,f,h).

To summarize, in HSA^LR^ mice, we identified changes in the levels of several markers related to the development of metabolic syndrome and insulin resistance observed in patients with DM1, including cholesterol, CPK, AST, LDH and blood glucose^79^. Interestingly, in HSA^LR^ mice expressing the CUG repeats only in muscle tissue^11^, we observed an impact on tissues beyond muscle, including plasma and liver markers. These observations indicate that HSA^LR^ mice are an excellent model for studying how altered muscle function originating from RNA toxicity can have a nonautonomous effect on other organs. Muscle tissue has a major role in whole-body metabolism^85,86^, and its dysfunction in DM1 leads progressively to a metabolic disease that could be studied in HSA^LR^ mice. While some effects of sex on disease symptoms, such as obesity and metabolic syndrome, seem partially mimicked in the HSA^LR^ mice, others are not. These discrepancies could result from the intrinsic limitation of HSA^LR^ mice that express the expanded CUG RNA only in skeletal muscles, and several aspects such as pain, gastrointestinal alterations or cognitive symptoms are not expected to be replicated in the model. The transgenic nature of the model probably impacts the extent to which sex-influenced parameters are recapitulated. Patients with DM1 carry the pathogenic CUG repeats in the *DMPK* transcript, while the HSA^LR^ mice model expresses the repeats in the 3′ UTR of the HSA transgene. The different genetic context, the absence of the *DMPK* background influencing repeat transcription and the fact that the expression of the repeats in this mouse model is limited to just skeletal muscle most likely represent intrinsic limitations of the HSA^LR^ model when it comes to biochemical parameters in blood plasma and related parameters, such as obesity.

## Conclusion

In summary, muscle disease in HSA^LR^ mice is comparable between males and females, which show similar increases in body weight, strength reduction and myotonia. Histologically, both sexes presented a dramatic increase in the percentage of fibers containing central nuclei, and this phenotype seemed to worsen with age. Analysis of the MBNL family of proteins showed similar changes in males and females (with the highest variability detected in the amount of MBNL1 protein). Likewise, analysis of missplicing events revealed comparable findings between sexes. Slight differences in misspicing events were found between male and female HSA^LR^ mice, which are negligible when compared with WT versus HSA^LR^ differences. Nevertheless, further research is warranted to determine whether other RNA-binding proteins reported to be altered in the HSA^LR^ model, such as CELF1 and Staufen1 (refs. ^43,87^), are similarly altered in males and females. In addition, it might be useful for further studies to characterize a broader, more in-depth set of missplicing events that could account for or relate to other disease phenotypes and abnormalities.

An overview of how all phenotypes examined in this study respond to the expression levels of the HSA transgene is shown in Supplementary Fig. 8, for all samples for which every parameter was measured. These correlations reveal how measured phenotypes behave differently in response to varying amounts of transgene expression. Whereas some parameters like myotonia and *Atp2a1* exon 22 inclusion displayed a sort of all-or-nothing effect (Supplementary Fig. 8b,l), others exhibited a more graded correlation with transgene expression, such as miR-218 relative expression and *Bin1* exon 11 inclusion (Supplementary Fig. 8h,m). Such differences indicate that for some parameters even small amounts of transgene expression are enough to induce DM1-like phenotypes in the HSA^LR^ model, while for other phenotypes, their severity depends on the levels of HSA expression. It must be noted that, for some phenotypes, reduced sample sizes made it difficult to recapitulate all findings, as was the case for most blood plasma markers (Supplementary Fig. 8o–t), for which most samples were not available for analysis of transgene expression.

Biochemical parameters are intrinsically variable according to sex, genotype and other factors such as age. This high variability made it challenging to reach consistent conclusions, and further studies should be performed to clarify the metabolic features of HSA^LR^ mice. Attention to sex and gender aspects in biomedical research has been a major initiative of the gender equality policy for research^88,89^. Females should, therefore, be included in biomedical research experimental designs, and their inclusion should be recommended in general guidelines for the use of laboratory animals^90^. Using HSA^LR^ females in DM1 studies has multiple advantages. Including females in preclinical and clinical assays can identify possible off-targets of the assayed therapies that could affect men and women differently^38,91^. For example, previous studies have reported differences in the degree of phenotype recovery between males and females following treatment with a combination of AMPK activators in HSA^LR^ mice^92^. Taken together with our observations that no major differences can be seen in the HSA^LR^ model between sexes in basal conditions, these previous findings suggest that differences between males and females might arise in the physiological response to future therapeutic candidates, further warranting the inclusion of females in future studies. Finally, experiments with HSA^LR^ using both sexes could help reduce the number of animals required in DM1 studies, which is in line with the 3R (reduce, replace and refine) principles. Therefore, we encourage researchers working with HSA^LR^ animals to include females in their studies and register and report their observations, which will contribute toward building a comprehensive understanding of DM1 disease in both sexes.

## Methods

### Animals

All parameters were studied in homozygous transgenic HSA^LR^ mice (line 20b), provided by Prof. C. Thornton (University of Rochester Medical Center, Rochester, NY, USA), and friend virus B (FVB) control mice, which provide the corresponding genetic background (named WT within this paper). Animals were housed under a 12-h light/12-h dark cycle with unrestricted access to food and water. Temperature and humidity were kept within the recommended range (20–24 °C and 40–60%, respectively).

All mouse handling and procedures followed the European law regarding laboratory animal care and experimentation (2003/65/CE) and were approved beforehand by Conselleria de Agricultura, Generalitat Valenciana. CEEA reference numbers for mice are as follows: 2016/VSC/PEA/0015, 2020/VSC/PEA/0164, 2020/VSC/PEA0166, 2020/VSC/PEA/0203, A1529567788818, A1458832800370, A20200902200603, A20200717201633, A20210218542822 and A20220322163151.

### Electromyography studies

Electromyography studies were performed blindly under general inhalation anesthesia (5% isoflurane for induction, 2% for maintenance) before euthanasia, as previously described^28^. Five needle insertions were made in each quadriceps muscle of both forelimbs, grading myotonia on a 0–10 scale: 0, no myotonia; 1–5, occasional myotonic discharge in <50% of total needle insertions; 6–7, myotonic discharge in >50% of total needle insertions; 8–9, myotonic discharge observed in nearly all of the insertions (80%); and 10, when the myotonic discharge occurred in all insertions.

### Grip strength test

The foreleg and hindleg grip strength test was performed blindly, using a Grip Strength Meter (BIO-GS3; Bioseb). The maximum pull force in grams was recorded on a digital force transducer when the mice grasped the bar, and the gauge of the force transducer was reset to 0 g after every measurement. The tension was recorded by the gauge at the time the mouse released its forepaws from the bar. Three consecutive measurements were taken for each mouse, letting the mouse rest for 30 s between each measurement. The body weight for each mouse was recorded in parallel and blindly, and used to normalize average grip strength. The data analyzed correspond to the average of the three measures. Both grip strength and grip strength normalized by weight were analyzed. We tested foreleg strength in the first set of animals, the same ones used to perform the whole phenotypical study (Fig. 1g). We measured both foreleg and hindleg strength in a second set of age-matched noneuthanized mice to confirm our observations in hindleg (Supplementary Fig. 3 and Supplementary Dataset 2).

### Tissue and sample collection

Tissue collection was performed after euthanasia. Each tissue of interest (that is, quadriceps and gastrocnemius) was appropriately collected and divided into two pieces. One of these pieces was immediately frozen in liquid nitrogen and later used for molecular studies, while the other was snap-frozen in liquid-nitrogen-chilled isopentane and then stored at −80 °C until tissue mounting for histology. Blood was collected by cardiac puncture exsanguination and collected into two different vials: a K3-EDTA vial (SARSTEDT, MICROVETTE 500, cat. no. 20.1341.100) or a vial treated with coagulation activators (KIMA MICRO TEST, cat. no. 811020). K3-EDTA vials were subjected to thorough shaking and −80 °C storage, while serum collection in vials containing coagulation activators needed 10 min centrifugation at 5,000 rpm, upon which supernatant (serum) was stored at −80 °C and the pellet discarded. Liver samples were used for animal genotyping.

### DNA extraction and genotyping

The genotype of every HSA^LR^ and FVB mouse used was confirmed by PCR analysis of genomic DNA from liver samples following the protocol described by Difranco et al.^91^, consisting of two PCRs with specific conditions and primers. Genomic DNA extraction was performed with the DNeasy Blood & Tissue kit (Qiagen, cat. no. 69504), following the manufacturer instructions but implementing modifications in the user-developed protocol ‘Purification of total DNA from soft tissues using the TissueLyser II (Qiagen) and the DNeasy® Blood &Tissue Kit’.

The first PCR reaction detects the presence of murine skeletal actin (MSA, *Acta1*) and the human skeletal actin transgene (HSA, *ACTA1*) using specific primers located in the 3′ noncoding region of both genes (MSA1, MSA2, HSA23 and HSA24 primers). A volume of 2 μL of genomic DNA (about 50 ng/μL) was analyzed in a 50 μL PCR containing 10 μL of 5× Colorless GoTaq Flexi Buffer (Promega), 5 μL of MgCl_2_ (25 mM), 1 μL of dNTPs (10 mM), 0,25 μL of GoTaq G2 DNA polymerase (Promega) and 1 μL each of the four primers described below. The GoTaq G2 DNA polymerase was activated at 95 °C for 2 min, followed by 28 cycles of denaturation at 95 °C for 30 s, annealing at 54.5 °C for 31 s and extension at 72 °C for 30 s. After the 28 cycles, DNA synthesis was completed by incubating the reaction at 72 °C for 5 min.

The second PCR reaction confirms the length of the CTG repeats (approximately 250) using primers that flanked the 5′ and 3′ ends of the repeat (HSA10 and HSA18 primers). In this case, 2 μL of genomic DNA (about 50 ng/μL) was analyzed in a 50 μL PCR containing 10 μL of 5× Green GoTaq Flexi Buffer (Promega), 5 μL of MgCl_2_ (25 mM), 1 μL of dNTPs (10 mM), 0,25 μL of GoTaq Flexi DNA Polymerase (Promega) and 1 μL each of the two primers described below. The GoTaq Flexi DNA polymerase was activated at 95 °C for 2 min and then submitted to 21 cycles of denaturation at 95 °C for 30 s, annealing at 64 °C for 1 min and extending at 72 °C for 2 min. After the 21 cycles, DNA synthesis was completed by incubating the reaction at 72 °C for 5 min.

All reactions were done on a DNA thermal cycler (either GeneAmp PCR System 9700, Applied Biosystems, or Gene Max Tc-s-B, Bioer). All reaction products were analyzed by electrophoresis through 1.7% agarose gels. A volume of 10 μl of 5× Green GoTaq Buffer (Promega) was added to the first PCR reaction products. The MSA1/MSA2 product is a 310 bp fragment, and the HSA23/HSA24 product, if positive, is a 249 bp fragment. If positive, the HSA10/HSA18 product is a fragment of around 1,200 bp (approximately 250 repeats).

Primer sequences used can be found in the Supplementary Information.

### Histological preparations and nucleus analysis

For quadriceps and gastrocnemius histology, muscles were embedded in OCT mounting medium (Leica) and 10-µm sections were obtained using a Leica CM1950 cryostat. Frozen sections were stained with the conventional hematoxylin and eosin protocol and mounted with DPX Mountant for histology (Sigma). Images were taken at 200× magnification with an Automated Upright LEICA DM4000 LED Microscope. The total number of fibers and the percentage of fibers containing central nuclei were quantified in 100 fibers per muscle. The number of mice analyzed per group (genotype × sex) can be found in Table 1. The total number of fibers and the fibers with central nuclei were counted using the ImageJ cell counter tool.

### Fluorescence in situ hybridization and immunofluorescence methods

Gastrocnemiu*s* and quadriceps muscles from five mice per group (genotype × sex) aged 4.5 months were used to analyze the foci presence and MBNL1 localization and intensity. Around seven sections were analyzed for each individual.

Localization of CUG^exp^ RNA (foci) by fluorescence in situ hybridization and localization of MBNL1 by immunofluorescence were carried out according to Bisset et al.^38^. In brief, 10-µm sections were obtained using a Leica CM1950 cryostat from both muscles embedded in OCT mounting medium. Sections were fixed in 4% paraformaldehyde for 15 min, washed with sterile phosphate-buffered saline (PBS) 1× and permeabilized with 0.5% Triton X-100/PBS for 5 min, all at room temperature.

For MBNL1 immunofluorescence, sections were blocked in 5% normal goat serum/PBS for 30 min at room temperature and incubated overnight at 4 °C with the primary antibody anti-MBNL1 (ab45899, 1/2,500 in 1% BSA/PBS). Then, sections were washed with PBS and incubated for 45 min in the dark at room temperature with the secondary antibody goat anti-rabbit Alexa Fluor 488 (A11008, Thermofisher, 1/400 in 1× PBS), washed with PBS and mounted with DAPI-containing VECTASHIELD mounting medium (Vector). MBNL1 signal was quantified using the ImageJ software, dividing green channel intensity by the muscle area. ImageJ software measures intensity and pixel size area from the confocal images.

For foci detection, sections were prehybridized in 30% formamide for 10 min at room temperature, hybridized with Cy3-(CAG)_7_-Cy3-labeled probe for 2 h in the dark at 37 °C (1:500, in hybridization buffer), posthybridized with formamide 30% for 30 min at 42 °C and then incubated with 1× SSC in 1× PBS for 30 min at room temperature and washed with PBS. Finally, sections were incubated with wheat germ agglutinin-FITC (1:400 in 1× PBS) for 45 min at room temperature to stain cell membranes, washed with PBS and mounted with DAPI-containing VECTASHIELD mounting medium (Vector). The percentage of ribonuclear foci was quantified with

$$\% \,{\mathrm{Foci}}=\frac{{\mathrm{Nuclei}}\,{\mathrm{with}}\,{\mathrm{foci}}}{{\mathrm{Total}}\,{\mathrm{nuclei}}}\times\,100.$$

Gastrocnemius foci images were also used to quantify the cross-sectional area of the fibers by the semi-automatic analysis tool of the ZEN software (Zeiss), which calculates the area of each fiber from the green channel. In both cases, images were acquired using the LSM800 confocal microscope (Zeiss) at 400× magnification.

### RNA extraction, RT-PCR, semiquantitative PCR and qRT–PCR

Total RNA from quadriceps and gastrocnemius muscles was extracted using the miRNeasy Mini Kit (Qiagen, cat. no. 217084) following the manufacturer’s instructions. For qRT–PCR detection of *Mbnl1* and *Mbnl2* transcripts, and alternative splicing measures by PCR, 1 µg of total RNA was digested with DNase I (Invitrogen, cat. no. 4716728001) and reverse transcribed using SuperScript II reverse transcriptase (Invitrogen, cat. no. 18064-014) and random hexanucleotides (Roche). Expression levels of *Mbnl1* and *Mbnl2*, as well as reference gene *Gapdh*, were determined by multiplex qPCR using the HOT FIREPol Multiplex qPCR Mix (Solisbiodyne #08-01-00001) and PrimeTime qPCR probe assays (IDT). Relative expression of *Mbnl1* and *Mbnl2* were measured and normalized to an endogenous control, *Gapdh*, using a 2^−ΔΔCt^ methodology. Primer and probe sequences used can be found in the Supplementary Information.

Alternative splicing was assayed by standard PCR, using 20 ng of cDNA as template, GoTaq polymerase (Promega, cat. no. M3008) and specific primers for *Atp2a1*, *Nfix*, *Clcn1*, *Mbnl1*, *Bin1* and *Cacna1s* alternative splicing events, as already described by Cerro-Herreros et al.^56^, Overby et al.^50^, Fugier et al.^70^ and Tang et al.^71^. The percentage of abnormal inclusion of the exons of each missplicing event was quantified using ImageJ. Levels of HSA transgene expression were determined by qRT–PCR, according to Wheeler et al.^40^.

For microRNA miR-218 detection by qPCR, 10 ng of total RNA was used for total microRNA retrotranscription using miRCURY LNA RT Kit (Qiagen, cat. no. 339340). A volume of 3 μL of diluted cDNA was used in the reaction with the miRCURY LNA SYBR Green PCR kit (Qiagen, cat. no. 339346) and Qiagen primers for qRT–PCR on the Applied Biosystems QuantStudio 5 Real-Time PCR System per the manufacturer’s protocol. Relative expression of hsa-miR-218-5p (Qiagen, cat. no. YP00206034) was measured and normalized to endogenous controls, RNU1A1 (Qiagen, cat. no. YP00203909) and U6 snRNA (Qiagen, cat. no. YP00203907) using the 2^−∆∆Ct^ method.

### Total protein extraction, quantitative dot blot and western blot

Mouse muscle samples were mechanically disaggregated with a TissueLyser II (Qiagen) and homogenized in RIPA Buffer (Thermo Scientific, cat. no. 89900) supplemented with protease and phosphatase inhibitors (Roche, cat. nos. 11873580001 and 4906845001). Total protein was quantified with Pierce BCA Protein Assay Kit (cat. no. 23225) using bovine serum albumin as the standard.

Immunodetection assay of MBNL1 and endogenous control tubulin was performed as described in Moreno et al.^93^. In brief, 2 µg/well of total protein, previously denatured at 100 °C for 5 min, was loaded in quantitative dot blot plates (Quanticision Diagnostics). Each sample was loaded in quadruplicate on three different dishes, of which one was incubated with mouse anti-MBNL1 antibody (1:200, MB1a(4A8), Developmental Studies Hybridoma Bank (DSHB)) and another with anti-α-tubulin antibody (1:1,000, 12G10, DSHB), at 4 °C overnight in both cases. Primary antibodies were detected using goat anti-mouse IgG (H + L) secondary antibody HRP-conjugated (1:3,500; Invitrogen). Immunoreaction was detected using Pierce ECL Western reagent (Thermo Scientific), and luminescence was acquired using an Infinite M200 PRO plate reader (Tecan).

Representative western blot was performed by denaturing 15 µg of samples for 5 min at 100 °C, protein separation by electrophoresis on 12% SDS–PAGE gels, transfer onto 0.45 µm nitrocellulose membranes (GE Healthcare) and blocking with 5% nonfat powdered milk in PBS-T (8 mM Na_2_HPO_4_, 150 mM NaCl, 2 mM KH_2_PO_4_, 3 mM KCl and 0.05% Tween 20, pH 7.4). Immunodetection of MBNL1 was achieved by incubating membranes at 4 °C with primary mouse anti-MBNL1 (1:200, MB1a(4A8), DSHB) and anti-Gapdh (1 h, 1:5,000, clone G-9, Santa Cruz) antibodies. Primary antibodies were detected with HRP-conjugated anti-mouse-IgG secondary antibody (1 h, 1:3,500, Sigma-Aldrich) and ECL Western Blotting Substrate (Pierce). Image acquisition was performed with an AMERSHAM ImageQuant 800 (GE Healthcare).

### Determination of biochemical parameters in blood plasma

Biochemical blood parameters were analyzed by Montoro Botella Laboratories. Using a Cobas 600 CCE Modular Analyzer (Roche), serum biochemistry profiles were obtained to evaluate total cholesterol, triglyceride levels, CPK, LDH, glucose and AST.

### Statistics

Phenotypes were measured in different sets of animals, euthanized on different dates, aged approximately 3–6 months; mice were individually identified. They were classified according to two factors: genotype (WT versus HSA^LR^) and sex (males versus females), and the age in months registered. Two different muscles were analyzed: quadriceps and gastrocnemius, and data available were merged for both muscles considered as duplicates; so, the *n* indicated in Table 1 and figure legends corresponds to the number of individuals, not the number of muscles. All data are summarized in Supplementary Dataset 1. Two-way ANOVA tests were performed for all phenotypes to detect to what extent they were defined by genotype, sex or interaction between them (genotype × sex). The data from the two muscles were included together in the model, but considering the animal as a source of error. Two-way ANOVA results are depicted in Supplementary Figs. 2 and 4–7. Boxplots (green for males and orange for females) represent the distribution of the phenotypes. Each colored dot corresponds to single measures performed in different muscles or in the whole organism (green for males and orange for females). Median values and sample sizes are described in Table 1. All statistic estimators, including the interquartile range, maximum and minimum, are detailed in Supplementary Dataset 3. The Wilcoxon nonparametric test was used to analyze further differences between groups (blue asterisk inside the plots). The measures of all phenotypes are also represented by the age of the animals (in months) in supplementary figures, classified by sex and genotype, and a tendency line is added to the dot graphs using a linear model. Three-way ANOVA was used to analyze the effect of age on the phenotypes studied. The single effect of sex, genotype and age was considered, as well as the interaction between these sources of variation; the results are presented in supplementary data. All statistics and graphics were performed using R-4.2.1 for Windows and Desktop RStudio-2022.02.3-492.

### Reporting summary

Further information on research design is available in the Nature Portfolio Reporting Summary linked to this article.

## Online content

Any methods, additional references, Nature Portfolio reporting summaries, source data, extended data, supplementary information, acknowledgements, peer review information; details of author contributions and competing interests; and statements of data and code availability are available at 10.1038/s41684-025-01506-7.

## Supplementary information

Supplementary Information Supplementary material of Methods section: primer list used for HSA transgene quantification and for qRT–PCR and Supplementary Figs. 1–8.

Reporting Summary

Supplementary Dataset 1 Raw data of all experiments in a matrix used for statistical analysis.

Supplementary Dataset 2 Foreleg and hindleg strength measures in a new set of mice.

Supplementary Dataset 3 Descriptive statistics of statistical analysis.

## Data Availability

The data that support the findings of this study are available from the corresponding author upon request.
