# Supplementary material for: Use of HSALR female mice as a model for the study of myotonic dystrophy type I
Source: Lab Anim (NY). 2025 Feb 27;54(4):92–102. doi: 10.1038/s41684-025-01506-7 (PMC11957995; doi:10.1038/s41684-025-01506-7)
Supplement: Supplementary file 1 — Supplementary material of Methods section: primer list used for HSA transgene quantification and for qRT–PCR and Supplementary Figs. 1–8. [file 41684_2025_1506_MOESM1_ESM.pdf]

lab animal

Supplementary information

https://doi.org/10.1038/s41684-025-01506-7

Use of HSA^{LR} female mice as a model for the study of myotonic dystrophy type I

In the format provided by the authors and unedited

Supplementary material

Methods: DNA extraction and genotyping.

Primers list for HSA transgene quantification.

PCR 1	MSA1 Primer	5′-TCCTCAGGACGACAATCGAC-3′
	MSA2 Primer	5′-CCTAAGGAGTTCACCCAGTCTG-3′
	HSA23 Primer	5′-AAACTTACATCTTCCCATGCTCC-3′
	HSA24 Primer	5′-GAGACGCCCTCTGAGAAACAG-3′
PCR 2	HSA10 Primer	5′-TCCACCGCAAATGCTTCTAGACACAC-3′
	HSA18 Primer	5′-GCAGGGGAGCATGGGAAGATGTAAG-3′

Methods: RNA extraction, RT-PCR, semiquantitative PCR and RT-qPCR.

Primers list for RT-qPCR.

Gapdh Probe	5’/5MAXN/-CGCCTGGTCACCAGGGCTGCT-/3BHQ_1/-3’
Gapdh Primer 1	5’- GAACGGATTTGGCCGTATTGG-3’
Gapdh Primer 2	5’- GATGGCAACAATCTCCACTTTGCC -3’
Mbnl1 Probe	5’-/56-FAM/TCGCAAATCAGCTGTGAGGAGATTCCCT/3IAbRQSp/-3’
Mbnl1 Primer 1	5’- TACCGATTGCACCACCAAAC -3’
Mbnl1 Primer 2	5’- GCTGCTTTCAGCAAAGTTGTC -3’
Mbnl2 Probe	5’-/56-FAM/CCCGGCAGACAGCACCATGATCGA/3IAbRQSp/-3’
Mbnl2 Primer 1	5’- GAGACAGACTGCCGCTTTG-3’
Mbnl2 Primer 2	5’- GGTTACGGTGTTGTCGTTTGT-3’

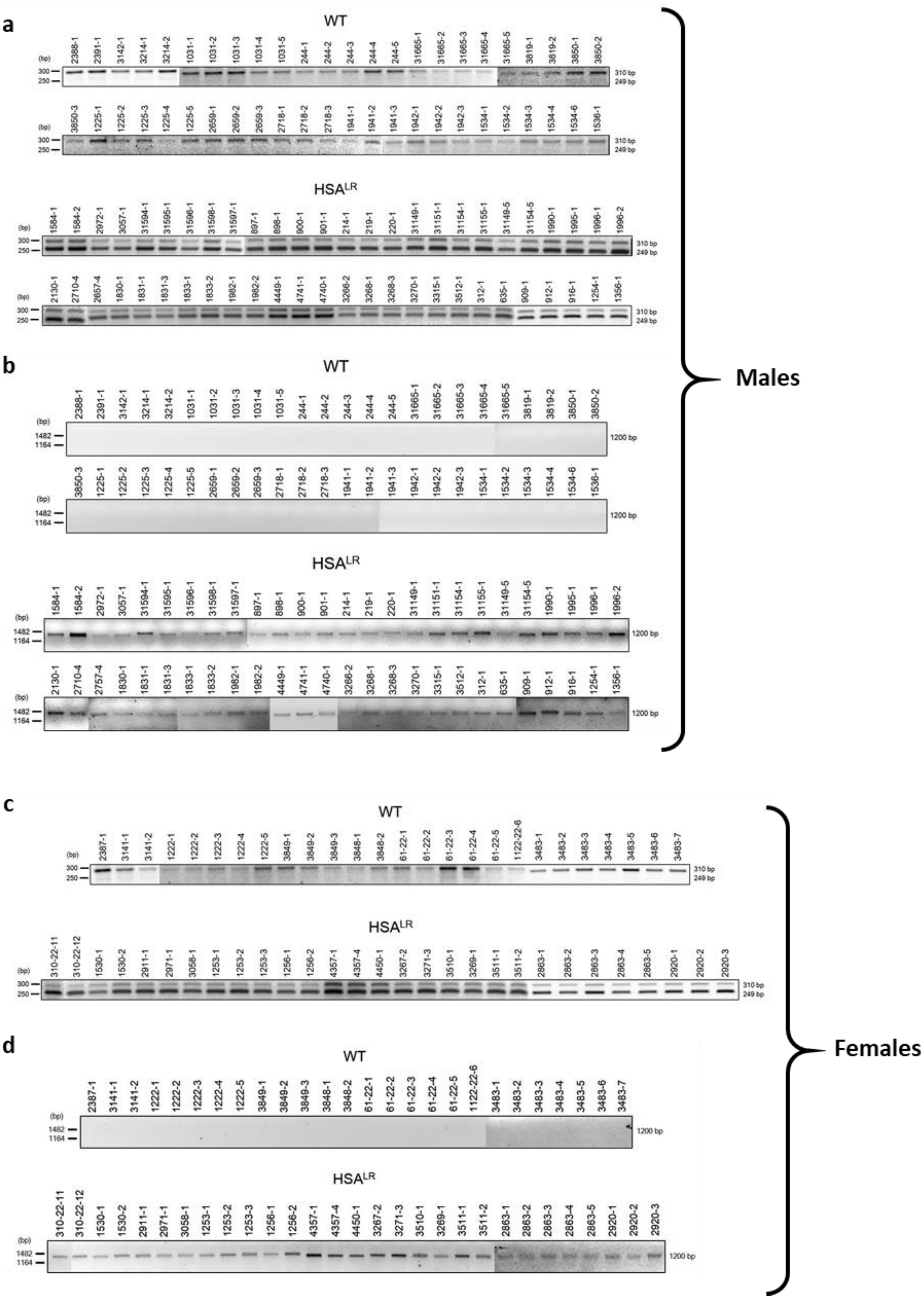

Supplementary Figure 1. Genotyping in 100 males (a, b) and 55 females (c, d) according to the protocol described in the methods. (**a, c**) The first PCR displays a single 310 bp band in controls and an extra 249 bp band in HSA^{LR}, corresponding to the human transgene. (**b, d**) The second PCR is negative in controls and amplifies a 1200 bp fragment in HSA^{LR} mice, corresponding to the CTG repeats.

a

Two-ways ANOVA	Source of variation (p-values)		
Phenotype	Genotype	Sex	Genot*Sex
HSA transgene expression	<2e-16 (***)	0.0184 (*)	0.0307 (*)
Myotonia	<2e-16 (***)	0.0504	0.2367
Weight [g]	8.37e-13 (***)	7.76e-06 (***)	0.286
Weight increase[fold]	7.27e-06 (***)	0.534	0.505
Forelimb strength	8.43e-16 (***)	0.984	0.139
Forelimb strength/ weight	2.40e-16 (***)	2.67e-05 (***)	0.828

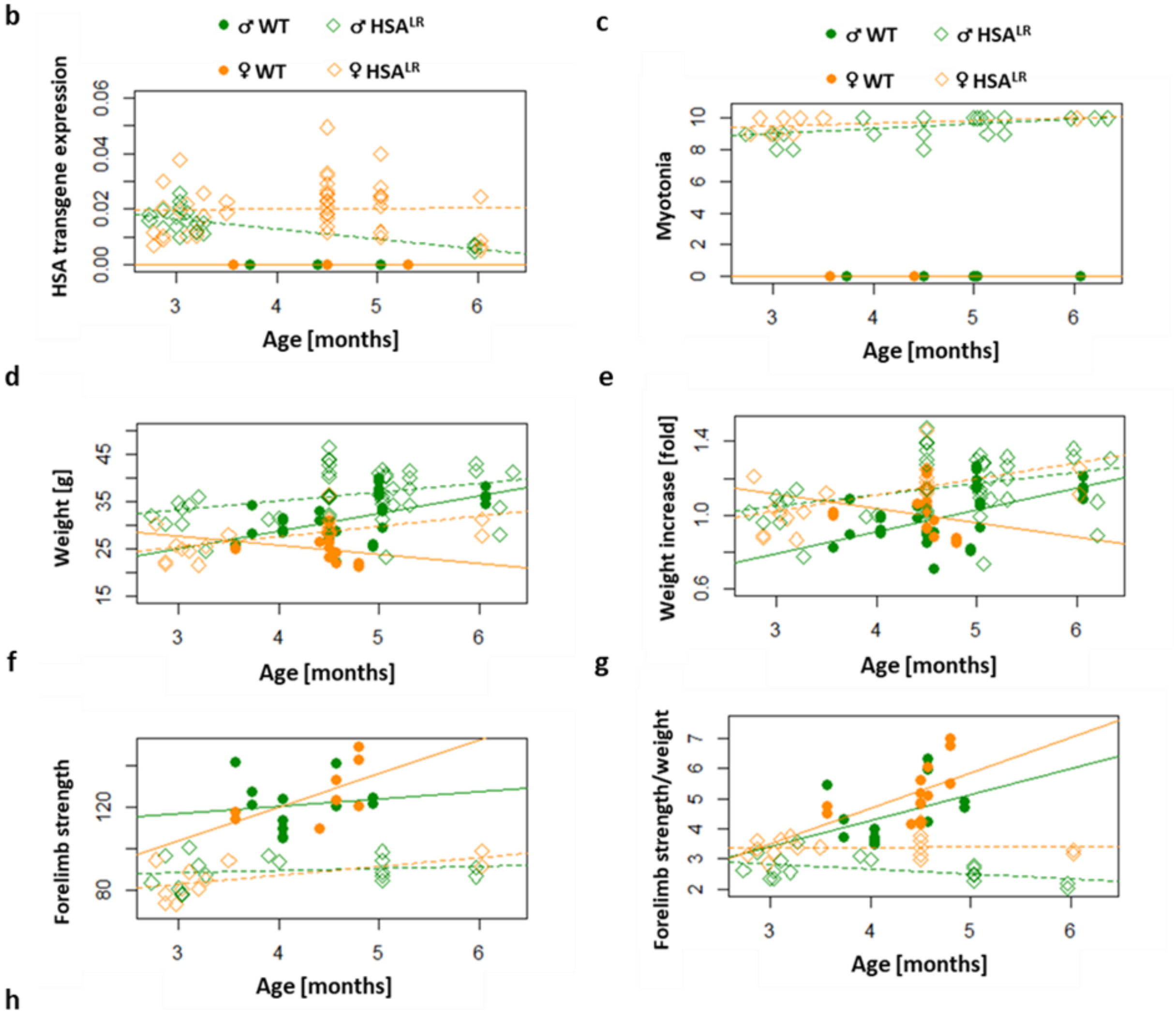

h

Three-ways ANOVA	Source of variation (p-values)						
Phenotype	Genotype	Sex	Age	Sex*Genot	Genot*age	Sex*age	Sex*Genot*age
HSA transgene expression	<2e-16 (***)	0.0158 (*)	0.1915	0.0166 (*)	0.4412	0.0260 (*)	0.2774
Myotonia	<2e-16 (***)	0.612622	0.000271 (***)	0.805263	0.057866	0.314276	0.836392
Weight [g]	1.17e-06 (***)	1.71e-14 (***)	5.30e-06 (***)	0.4648	0.3451	0.5356	0.0771
Weight increase[fold]	1.01e-06 (***)	0.494	4.97e-06 (***)	0.772	0.861	0.434	0.046 (*)
Forelimb strength	7.48e-16 (***)	0.9836	0.0306 (*)	0.2341	0.1908	0.1825	0.3555
Forelimb strength/ weight	<2e-16 (***)	6.93e-06 (***)	0.873087	0.824913	0.000515 (***)	0.184276	0.919766

Supplementary Figure 2. Organismal-level phenotypes analysis (a) Statistical analysis of organismal-level phenotypes. ANOVA two-way separates the effect of sex (males vs females) and Genotype (Control vs HSA^{LR}) and detects interaction between them (Genot*Sex). **(b-g)** Data from **Figure 1** is represented according to the age of the animals. Tendency lines are drawn: regular lines correspond to control animals, and dotted lines correspond to HSA^{LR} animals. **(h)** Three-way ANOVA (Genotype, Sex and Age) is performed to detect the influence of age on the phenotypes. [***: p< 0.001; **: p< 0.01; *: p< 0.05].

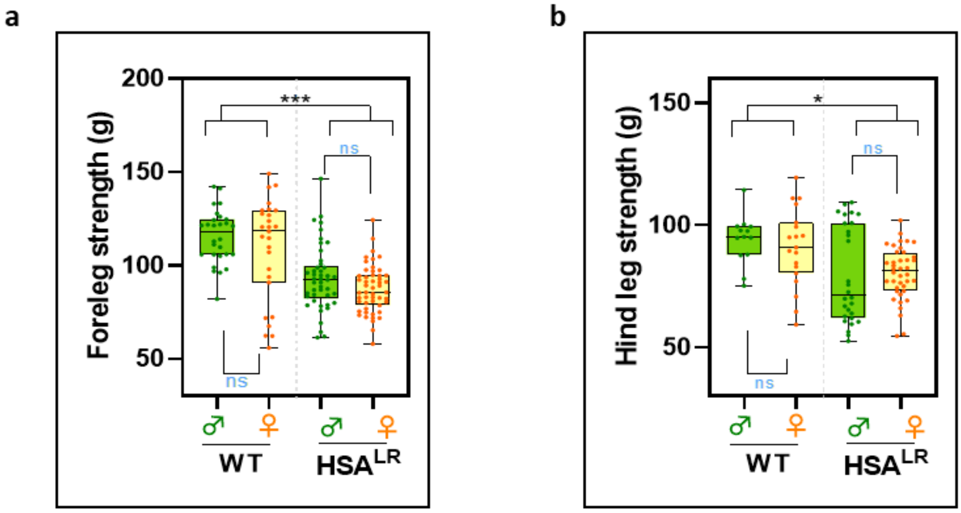

c

	Males WT		Females WT		Males HSA^{LR}		Females HSA^{LR}	
**Phenotype**	**Average age (months)**	**n**	**Average age (months)**	**n**	**Average age (months)**	**n**	**Average age (months)**	**n**
Foreleg strength	3,7	28	3,8	27	3,8	45	4,2	51
Hind leg strength	3,4	14	3,9	19	3,5	28	4,2	40

Supplementary Figure 3. Functional analysis. Analysis of foreleg **(a)** and hindleg **(b)** of a larger set of young mice with an average age of 3,4 to 4,2 months **(c)** confirms observations presented in **Figure 1**. [***: p< 0.001; *: p< 0.05; ns: non-significant].

a

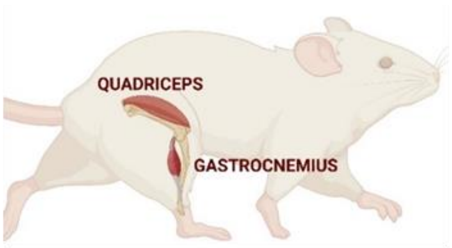

b

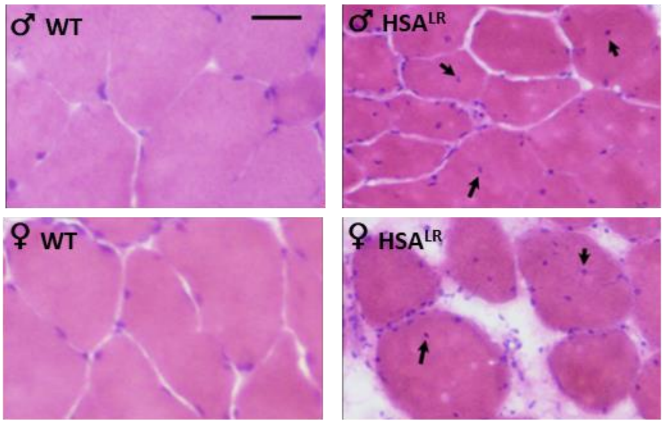

c

Two-ways ANOVA	Source of variation (p-values)		
Phenotype	Genotype	Sex	Genot*Sex
% fibers w/central nuclei	<2e-16 (***)	0.188	0.255
% nuclei w/foci	1.08e-15 (***)	0.20442	0.0163 (*)
Mbnl1 signal intensity	0.913	0.381	0.182
fiber area (μm^2)	0.103	0.881	0.511

d

Three-ways ANOVA	Source of variation (p-values)						
Phenotype	Genotype	Sex	Age	Sex*Genot	Genot*age	Sex*age	Sex*Genot*age
% fibers w/central nuclei	<2e-16 (***)	0.15227	3.87e-07 (***)	0.27029	0.00685	0.05231	0.30294

e

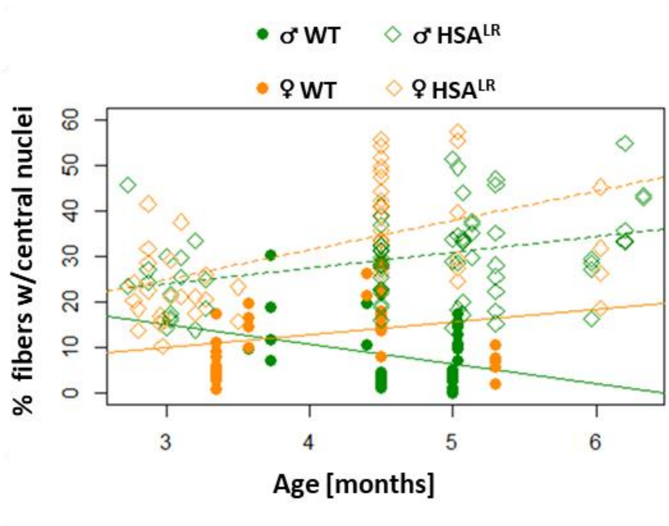

f

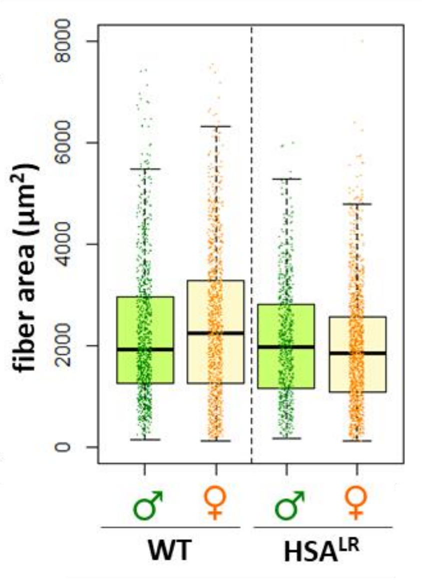

Supplementary Figure 4. Muscle fiber analysis. (a) Anatomical disposition of muscles studied: *quadriceps* and *gastrocnemius* (created with BioRender) **(b)** Bright-field microscopy pictures (200X magnification; scale bar = 50 µm) of hematoxylin/eosin staining of *gastrocnemius* sections show an increased amount of central nuclei (black arrows).. (**c**) Statistical analysis of histology phenotypes. Two-way ANOVA shows that the effect of sex and the interaction Genot*Sex is significant for the presence of foci and intensity of Mbnl1 signal. [***: p< 0.001; **: p< 0.01; *: p< 0.05] **(d)** Three-way ANOVA (Genotype, Sex and Age) is performed to detect the influence of age on the percentage of fibers with central nuclei [***: p< 0.001] **(e)** The percentage of fibers with central nuclei in *gastrocnemius* and *quadriceps* according to age. **(f)** Fiber area measured in sections of 20 animals (n=5 per group, 4,5 months old).

a

Two-ways ANOVA	Source of variation (p-values)		
Phenotype	Genotype	Sex	Genot*Sex
Mbnl1 mRNA	5.83e-08 (***)	0.548	0.544
Mbnl2 mRNA	1.38e-06 (***)	0.782	0.960
Mbnl1 protein	0.0121 (*)	0.8944	0.8273
miR-218	2.55e-06 (***)	0.501	0.103

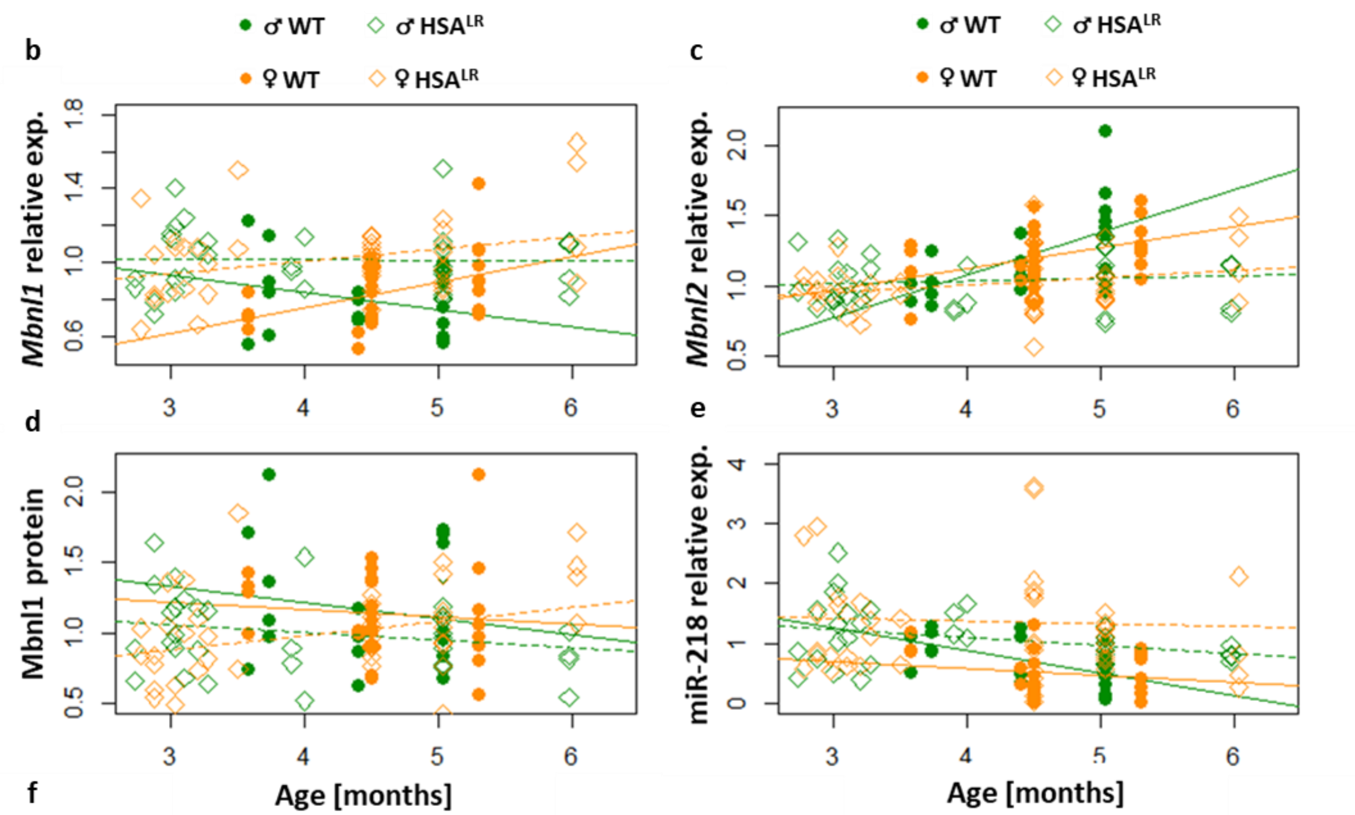

f

Three-ways ANOVA	Source of variation (p-values)						
Phenotype	Genotype	Sex	Age	Sex*Genot	Genot*age	Sex*age	Sex*Genot*age
Mbnl1 mRNA	2.37e-08 (***)	0.5342	0.0607	0.5242	0.9924	0.0101 (*)	0.1082
Mbnl2 mRNA	2.63e-07 (***)	0.76654	0.00189 (**)	0.94560	0.00128 (**)	0.89021	0.10181
Mbnl1 protein	0.0112 (*)	0.8931	0.7141	0.8245	0.1847	0.0295 (*)	0.5929
miR-218	2.81e-06 (***)	0.502	0.157	0.105	0.491	0.464	0.690

Supplementary Figure 5. Muscleblind family analysis. (a) ANOVA two-way shows that phenotypes analyzed are strongly determined by Genotype (Ctrl vs. HSA^{LR}) but not by sex. (**b-e**) Data from Figure 3 are represented according to the age of the animals. Trend lines are drawn: regular lines correspond to control animals, and dotted lines correspond to HSA^{LR} animals. (**f**) Three-way ANOVA (Genotype, Sex and Age) is performed to detect the influence of age on the phenotypes [***: p< 0.001; **: p< 0.01; *: p< 0.05].

a

Two-ways ANOVA	Source of variation (p-values)		
Phenotype	Genotype	Sex	Genot*Sex
Nfix % ex7 inclusion	<2e-16 (***)	0.380	0.301
Mbnl1 % ex5 inclusion	<2e-16 (***)	0.0195 (*)	0.2661
Clcn1 % ex7a inclusion	<2e-16 (***)	0.575	0.284
Atp2a1 % ex22 inclusion	<2e-16 (***)	0.0077 (**)	0.08134
Bin1 % ex11 inclusion	<2e-16 (***)	0.846	0.942
Cacna1s ex29 inclusion	<2e-16 (***)	0.0784	0.2115

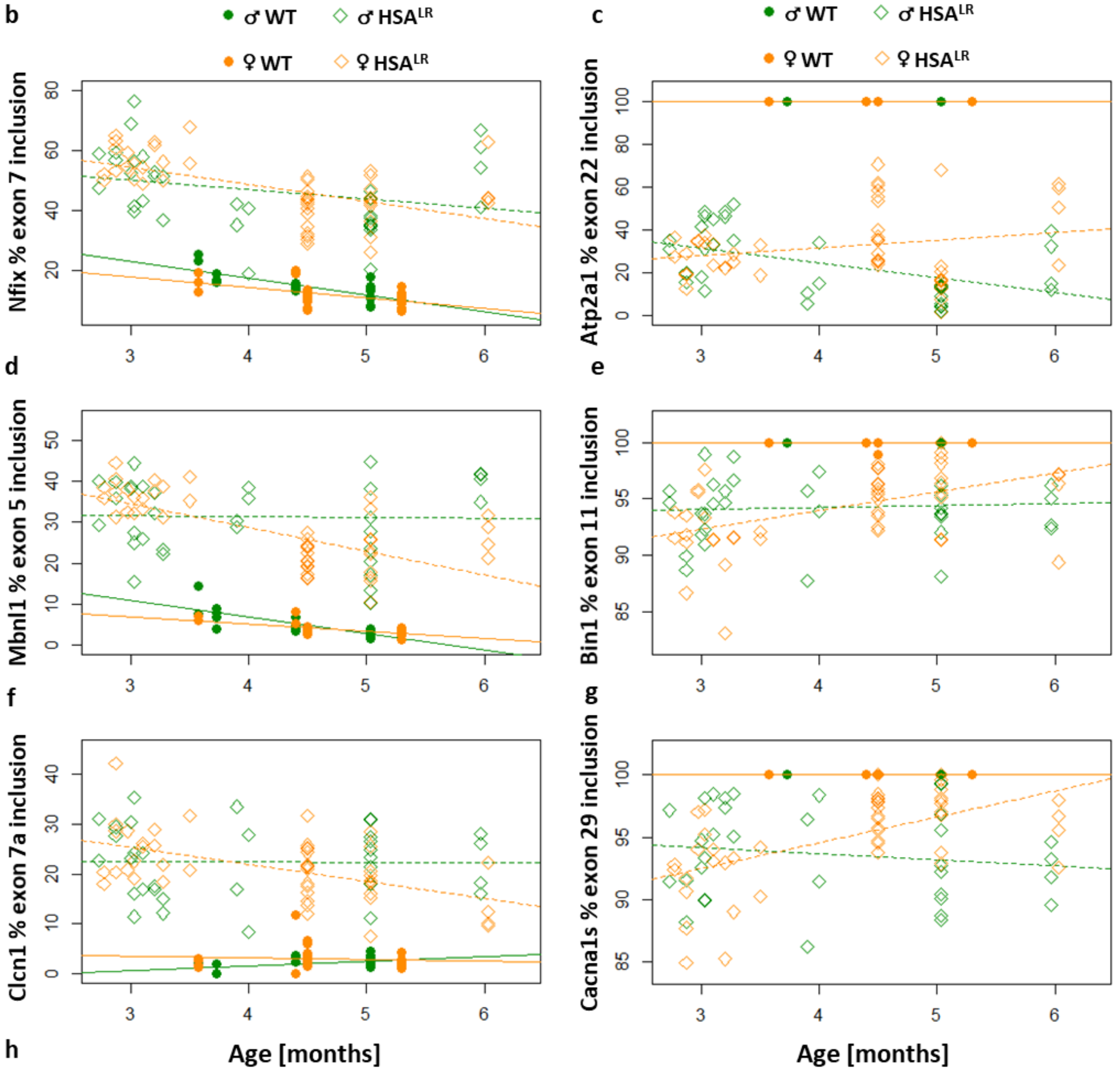

h

Three-ways ANOVA	Source of variation (p-values)						
Phenotype	Genotype	Sex	Age	Sex*Genot	Genot*age	Sex*age	Sex*Genot*age
Nfix % ex7 inclusion	<2e-16 (***)	0.335	5.63e-07 (***)	0.279	0.921	0.270	0.312
Mbnl1 % ex5 inclusion	<2e-16 (***)	0.007706 (**)	1.82e-06 (***)	0.198557	0.702636	0.0005 (***)	0.022046 (*)
Clcn1 % ex7a inclusion	<2e-16 (***)	0.55217	0.00378 (**)	0.25265	0.12176	0.00430 (**)	0.47200
Atp2a1 % ex22 inclusion	<2e-16 (***)	0.004772 (**)	0.304894	0.065142	0.75687	0.00026 (***)	0.111360
Bin1 % ex11 inclusion	<2e-16 (***)	0.834939	0.000835 (***)	0.930813	0.00912 (**)	0.12199	0.255698
Cacna1s ex29 inclusion	<2e-16 (***)	0.05353	0.00581 (**)	0.16789	5.94e-05 (***)	0.17588	0.07455

Supplementary Figure 6. Analysis of alternative splicing defects (a) Two-way ANOVA analysis shows that genotype determines the muscle splicing pattern; minor effects are observed due to the sex of the animals. **(b-g)** Data from Figure 4 are represented according to the age of the animals. Trend lines are drawn: regular lines correspond to control animals, and dotted lines correspond to HSA^{LR} animals. **(h)** Three-way ANOVA (Genotype, Sex and Age) is performed to detect the influence of age on the phenotypes. [***: p< 0.001; **: p< 0.01; *: p< 0.05].

a

Two-ways ANOVA	Source of variation (p-values)		
Phenotype	Genotype	Sex	Genot*Sex
Cholesterol (mg/dL)	0.00264 **	4.8e-06 ***	0.11321
Triglycerides (mg/dL)	0.829	0.125	0.305
CPK (U/L)	0.00666 **	0.91482	0.22161
LDH (U/L)	0.000114 ***	0.006738 **	4e-05 ***
Glucose (mg/dL)	0.2338	0.0132 *	0.0107 *
AST (U/L)	0.000814 ***	0.253388	0.130614

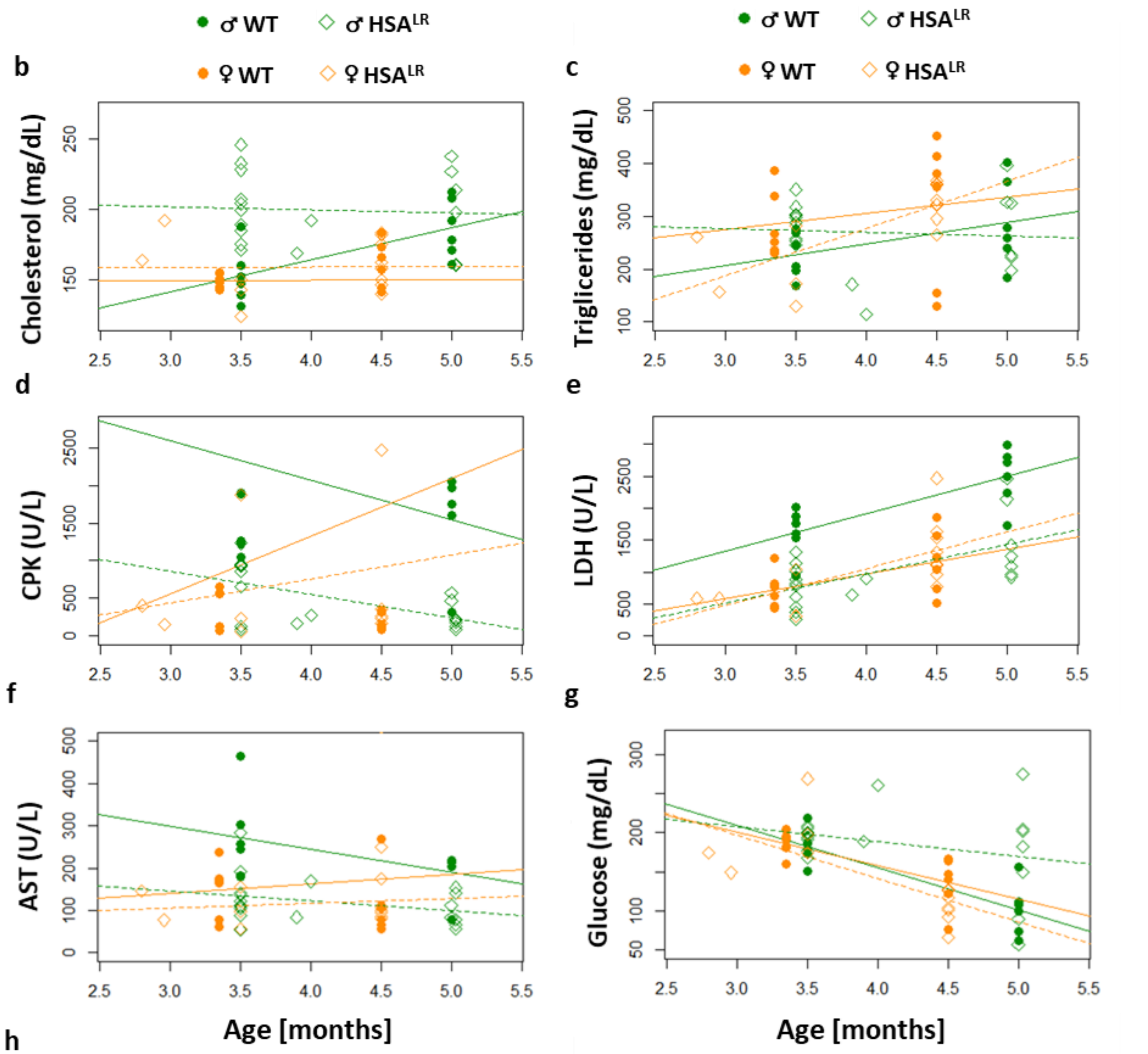

h

Three-ways ANOVA	Source of variation (p-values)						
Phenotype	Genotype	Sex	Age	Sex*Genot	Genot*age	Sex*age	Sex*Genot*age
Cholesterol (mg/dL)	0.00253 (**)	2.3e-06 (***)	0.31930	0.10699	0.10569	0.42091	0.20645
Trigliceriedes (mg/dL)	0.7652	0.1817	0.0446 (*)	0.2922	0.1032	0.7877	0.0902
CPK (U/L)	0.00673 (**)	0.91754	0.88709	0.22090	0.94978	0.09707	0.54369
LDH (U/L)	7.60e-05 (***)	0.000809 (***)	1.14e-07 (***)	6.01e-05 (***)	0.978031	0.903693	0.391266
Glucose (mg/dL)	0.15990	0.05721	1.9e-06 (***)	0.00978 (**)	0.26681	0.30702	0.14073
AST (U/L)	0.000947 (***)	0.291094	0.374340	0.131598	0.659452	0.139528	0.541457

Supplementary Figure 7. Analysis of plasma biochemical parameters (a) Two-way ANOVA summary of plasma parameters. **(b-g)** Data from Figure 5 are represented according to the age of the animals. Tendency lines are drawn: regular lines correspond to control animals, and dotted lines correspond to HSALR animals. **(h)** Three-way ANOVA (Genotype, Sex and Age) is performed to detect the influence of age on the phenotypes. [***: p< 0.001; **: p< 0.01; *: p< 0.05].

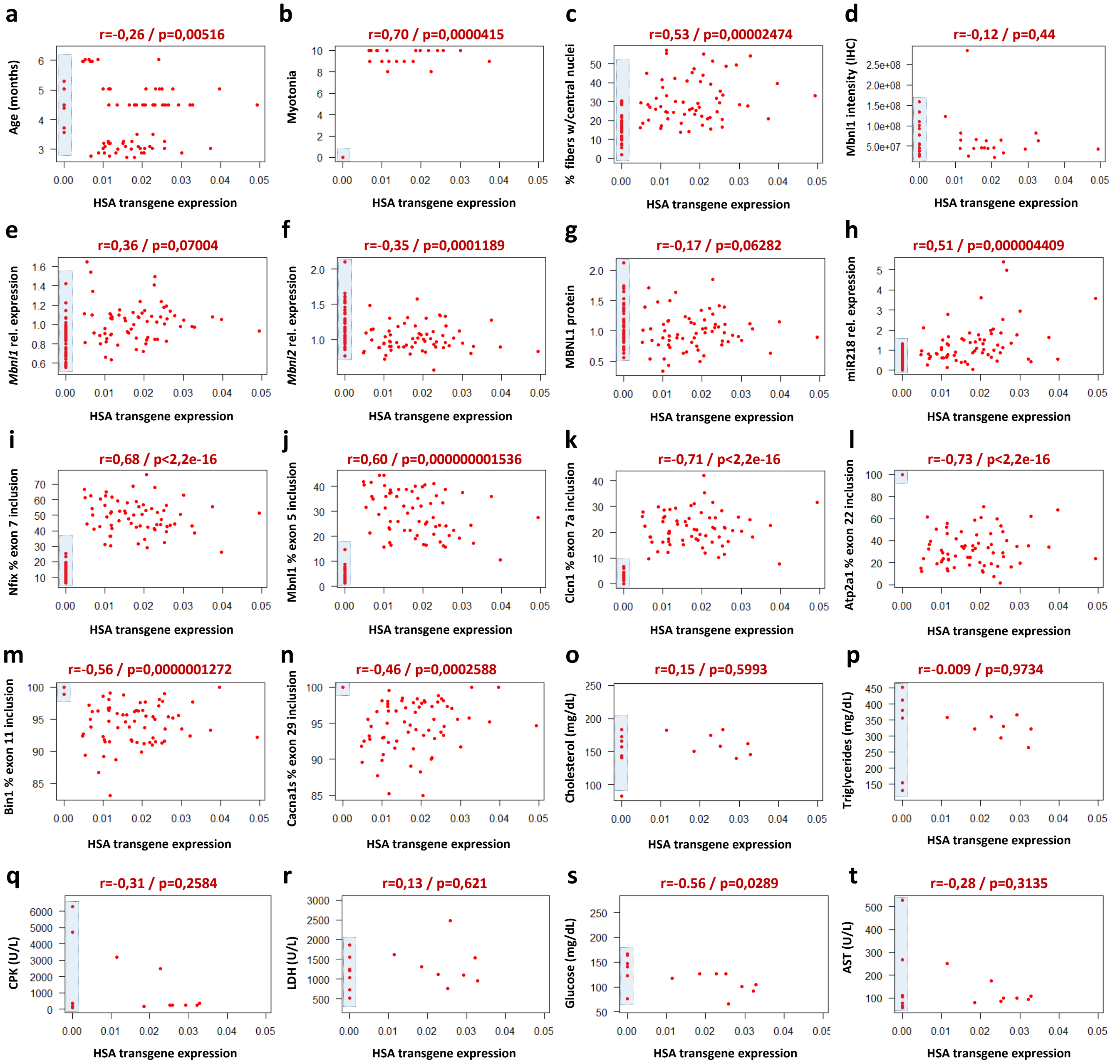

Supplementary Figure 8. Correlation analysis. Correlations of the different phenotypes studied in this paper with the presence of the *HSA* transgene: (**a**) age; **(b)** myotonia; **(c)** percentage of fibers with central nuclei; **(d)** Mbnl1 intensity; **(e)** *Mbnl1* relative expression; **(f)** *Mbnl2* relative expression; **(g)** MBNL1 protein; **(h)** *miR-218* relative expression; percentage of abnormal inclusion of **(i)** exon 7 in *Nfix*, **(j)** exon 5 in *Mbnl1*, **(k)** exon 7a in *Clcn1*; percentage of abnormal exclusion of (**l**) exon 22 in *Atp2A1*, **(m)** exon 11 in *Bin1*, **(n)** exon 29 in *Cacna1s*; **(o)** cholesterol levels; **(p)** triglycerides levels; **(q)** CPK levels; **(r)** LDH levels; **(s)** glucose levels; and **(t)** AST levels. Data from WT mice, which do not carry the *HSA* transgene, are marked in blue boxes.
